# Co-Evolution of Transcriptional Silencing Proteins and the DNA Elements Specifying Their Assembly

**DOI:** 10.1371/journal.pbio.1000550

**Published:** 2010-11-30

**Authors:** Oliver A. Zill, Devin Scannell, Leonid Teytelman, Jasper Rine

**Affiliations:** 1 Department of Molecular and Cell Biology, and California Institute for Quantitative Biosciences, University of California–Berkeley, Berkeley, California, United States of America; 2 Department of Biology, Massachusetts Institute of Technology, Cambridge, MA, United States of America; University of Nottingham, United Kingdom

## Abstract

Co-evolution of transcriptional regulatory proteins and their sites of action has been often hypothesized but rarely demonstrated. Here we provide experimental evidence of such co-evolution in yeast silent chromatin, a finding that emerged from studies of hybrids formed between two closely related *Saccharomyces* species. A unidirectional silencing incompatibility between *S. cerevisiae* and *S. bayanus* led to a key discovery: asymmetrical complementation of divergent orthologs of the silent chromatin component Sir4. In *S. cerevisiae*/*S. bayanus* interspecies hybrids, ChIP-Seq analysis revealed a restriction against *S. cerevisiae* Sir4 associating with most *S. bayanus* silenced regions; in contrast, *S. bayanus* Sir4 associated with *S. cerevisiae* silenced loci to an even greater degree than did *S. cerevisiae*'s own Sir4. Functional changes in silencer sequences paralleled changes in Sir4 sequence and a reduction in Sir1 family members in *S. cerevisiae*. Critically, species-specific silencing of the *S. bayanus HMR* locus could be reconstituted in *S. cerevisiae* by co-transfer of the *S. bayanus* Sir4 and Kos3 (the ancestral relative of Sir1) proteins. As Sir1/Kos3 and Sir4 bind conserved silencer-binding proteins, but not specific DNA sequences, these rapidly evolving proteins served to interpret differences in the two species' silencers presumably involving emergent features created by the regulatory proteins that bind sequences within silencers. The results presented here, and in particular the high resolution ChIP-Seq localization of the Sir4 protein, provided unanticipated insights into the mechanism of silent chromatin assembly in yeast.

## Abstract 2

As shown by genetic assays in *Saccharomyces* interspecies hybrids, the co-evolution of heterochromatin assembly proteins with silencer elements allows transcriptional silencing functions to be maintained in rapidly evolving regions of the genome.

## Author Summary

As eukaryotic species evolve, transcriptionally silent portions of their genomes—termed “heterochromatin”—mutate rapidly. To maintain the “off” state of certain genes in silenced regions, regulatory DNA sequences called silencers, which reside within a rapidly mutating region, must co-evolve with the regulatory proteins that bind these sequences to turn off transcription. Although hypothesized to occur widely in nature, such “molecular co-evolution” of genetic regulators has been demonstrated in only a few cases. Unlike previous examples of gene regulatory co-evolution, we found that the transcription factors that bind silencers in two budding yeast species are, in fact, functionally interchangeable, even though the silencers are not. Surprisingly, the Sir1 and Sir4 silencing proteins, which are heterochromatin components that bind the transcription factors rather than the silencer DNA sequences per se, are the proteins engaged in rapid co-evolution with the silencers. Silencer sequences therefore contain additional, evolutionarily labile information directing the assembly of heterochromatin. As mutations in Sir1 and Sir4 over evolutionary time can compensate for changes in the silencers, this “extra information” likely involves cooperative assembly of the transcription factors with the Sir1 and Sir4 “adaptor” proteins. The localization patterns of two species' Sir4 proteins across both species' genomes in interspecies yeast hybrids illuminate unexpected features of heterochromatin structure and assembly.

## Introduction

Among all specialized chromatin structures, the difference between heterochromatin and euchromatin is perhaps the most fundamental, motivating intense study of the differences between these two structures. DNA sequences within heterochromatic regions evolve rapidly in animals [1],[2], plants [3],[4], and fungi [5], presenting a paradox of how the specification of heterochromatin structure persists despite rapid changes in the underlying sequence [6]. In *Saccharomyces* the biology of heterochromatin has proven eminently accessible to genetic studies through its role in gene silencing [7], and comparative studies of silencing now seem poised to illuminate key processes underlying heterochromatin evolution.

Molecular co-evolution of transcriptional regulatory proteins with their sites of action has been proposed to maintain regulatory functions across species divergence [8],[9]. In this context, “co-evolution” is typically understood as compensatory changes in a DNA sequence motif and the DNA-binding domain of the cognate transcription factor. Although it has been suggested that such co-evolution is prevalent in nature [8], in only a few instances has it been directly tested [10]–[12]. In Dipteran insects, for example, co-evolution of *bicoid* binding sites in the *hunchback* promoter and the *bicoid* homeodomain has been proposed to maintain *hunchback*-mediated developmental patterning along the anterior/posterior axis in *Musca* and *Drosophila* [13],[14]. However, the large size and complexity of animal regulatory elements, and the difficulty of performing cross-species complementation tests in animals, have precluded clear distinction between regulatory divergence and bona fide co-evolution.

Transcriptional silencing by Sir (Silent Information Regulator) proteins is necessary for the specialized haploid mating-type system found in *Saccharomyces* [15],[16]. DNA regulatory elements termed “silencers” contain binding sites for the Origin Recognition Complex (ORC), Rap1, and Abf1, which in turn direct the assembly of silent chromatin structures at the *HML* and *HMR* loci. The current model for the establishment of silencing holds that a Sir2/Sir3/Sir4 complex is brought to silencers by protein-protein interactions between ORC and Sir1, and between Rap1 and Sir4 [7]. Upon nucleation of these complexes, silent chromatin formation is catalyzed by the histone deacetylase activity of Sir2, and propagated, at least in part, through interactions between Sir3 and newly deacetylated histone tails [17]–[19]. Sir proteins are not thought to bind specific DNA sites; instead, efficient nucleation of silencing complexes at silencers requires interactions between Sir1 and Sir4, bridging the ORC-Sir1 and Rap1-Sir4 interactions [20]. Silencing also occurs at telomeres, which recruit Sir proteins primarily through arrays of Rap1 binding sites within the terminal repeats (TG_1–3_) [21]. However, more complex regulatory elements reside near the terminal repeats, and at some telomeres these may also serve to recruit Sir proteins [32],[23].

**Figure 1:**
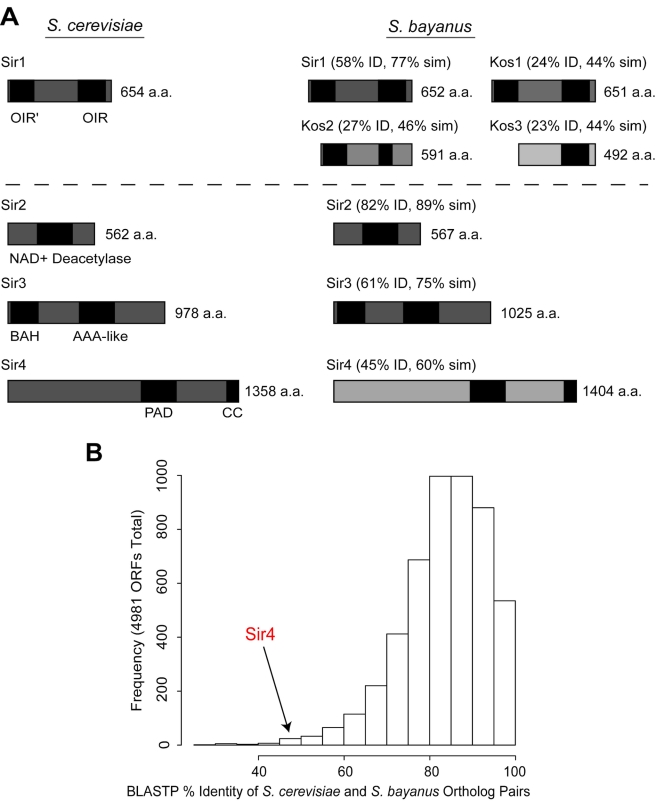
Comparative analysis of Sir proteins in *S. cerevisiae* and *S. bayanus*. (A) Comparison of the Sir protein complements of *S. cerevisiae* and *S. bayanus*. Percent identity (ID) and similarity (sim) for each orthologous pair of proteins, as determined by BLASTP alignments, is indicated above each *S. bayanus* ortholog. Protein lengths in numbers of amino acids (a.a.) are given to the right of each schematic. Black boxes indicate known domains within each protein (to approximate scale), with domain names indicated below the *S. cerevisiae* orthologs. OIR, ORC-Interacting Region; BAH, Bromo-Adjacent Homology domain; PAD, Partitioning and Anchoring Domain; CC, Coiled-Coil. (B) Percent identities of orthologous *S. cerevisiae* and *S. bayanus* proteins as reported by BLASTP. Histogram shows percent identities of orthologous *S. cerevisiae* and *S. bayanus* proteins based on BLASTP alignments. The distribution of percent identity of orthologous protein pairs, in bins of 5 percent increments, is plotted versus the number of orthologous pairs in each bin. The bin containing Sir4 (45% identity) is indicated with an arrow.

We have recently shown that silencer elements are among the most rapidly evolving regulatory sequences in *Saccharomyces* genomes [5]; however, the regulatory proteins that directly bind silencers are highly conserved, essential proteins. Intriguingly, the Sir1 and Sir4 proteins parallel the silencers in their rapid evolution, but these proteins show distinct patterns of evolution. *SIR1*-related genes have undergone multiple duplication and loss events: for example, *S. bayanus* has four functional paralogs of the single *S. cerevisiae SIR1* gene, including the ancestral *KOS3* (Kin Of Sir1) paralog, which *S. cerevisiae* has lost along with two other paralogs (Figure 1A) [24]. In contrast, the Sir4 protein is among the 40 most diverged proteins between *S. cerevisiae* and *S. bayanus* (Figure 1B), with 45% identity between its orthologs relative to a genome-wide average of 83% identity [25],[26]. Although *SIR2* and *SIR3* each have a paralog resulting from the whole-genome duplication (*SIR2* has three additional, more ancient paralogs), neither gene has experienced subsequent duplication or loss events [27],[28].

*S. cerevisiae* and *S. bayanus* are post-zygotically isolated—haploids of these two species can mate to form mitotically stable hybrid diploids, but meiotic spores derived from these diploids are usually inviable [29],[30]. The rapid evolution of the silencers, the Sir4 protein sequence, and the elaboration of Sir1 paralogs make these two species an excellent phylogenetic context for comparative studies of silencing. Here, we describe functional studies in *S. cerevisiae/S. bayanus* interspecies hybrids that demonstrated how co-evolution among two heterochromatin proteins, Sir1 and Sir4, and multiple silencer DNA elements allowed two divergent lineages to maintain robust silencing despite these rapid genetic changes. This example of regulatory co-evolution is of particular interest because the co-evolving proteins are not the agents that directly bind to the divergent regulatory DNA sites.

## Results

### An Incompatibility between *S. cerevisiae SIR4* and *S. bayanus HMR* Revealed by Genetic Analysis of Interspecies Hybrids

In the course of a genetic screen for *S. bayanus* silencing mutants, we discovered that *S. cerevisiae SIR4* failed to complement *S. bayanus sir4Δ* mutants for silencing of both *HML* and *HMR*, but *S. cerevisiae SIR2* and *SIR3* complemented mutations in *S. bayanus* orthologs (Figure S1; Zill et al. in preparation). This result was unanticipated as there are many cases of human proteins that can replace their yeast counterparts, even for proteins that function in large complexes and have considerably more sequence divergence than that seen between *S. cerevisiae* and *S. bayanus* proteins [31]–[34]. The incompatibility was unidirectional as *S. bayanus SIR2*, *SIR3*, and *SIR4* complemented *S. cerevisiae sir2Δ*, *sir3Δ*, and *sir4Δ*, respectively.

**Figure 2:**
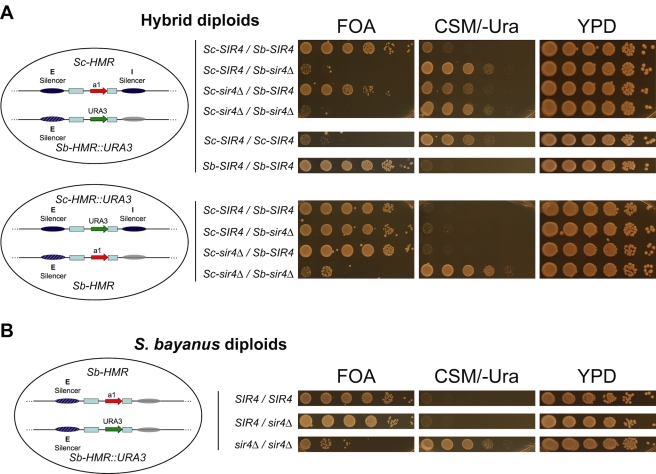
Incompatibility between *S. cerevisiae SIR4* and *S. bayanus HMR* in *S. cerevisiae/S. bayanus* interspecies hybrids. (A) Silencing of the *Sb-HMR::URA3* reporter gene (top panel) or the *Sc-HMR::URA3* reporter gene (bottom panel) in *S. cerevisiae/S. bayanus* hybrids was assayed by growth on selective media. For each strain, a 10-fold dilution series of yeast cells was spotted onto medium counter-selective for *URA3* expression (FOA), selective for *URA3* expression (CSM/-Ura), or rich medium (YPD). Schematics at left show the configurations of the salient features of two species' *HMR* loci in each hybrid strain: silencers (ovals), mating-type cassette homology regions (blue boxes), *HMR* **a**1 ORF (red arrow), and *URA3* ORF (green arrow). Gray oval indicates the presumed location of the *Sb-HMR-I* silencer. Presence or absence (*Δ*) of the *S. cerevisiae* (*Sc*) and *S. bayanus* (*Sb*) *SIR4* alleles are indicated to the right of schematics. See Table S1 for complete strain genotypes. (B) Silencing of the *Sb-HMR::URA3* reporter gene in wild-type, *SIR4/sir4Δ*, or *sir4Δ/sir4Δ S. bayanus* diploids.

Importantly, *SIR4* functional divergence was due to one or more coding changes, as the level of expression of the two Sir4 orthologs, measured at either the RNA or protein level, was equivalent (Figure S2). To assay the function of both species' silencing machineries in the same cellular milieu, we developed a highly sensitive transcriptional reporter assay in *S. cerevisiae*/*S. bayanus* interspecies hybrid diploids that allowed us to monitor silencing of each species' *HMR* locus (hereafter referred to as *Sc-HMR* or *Sb-HMR*). The reporter consisted of the *K. lactis URA3* open reading frame placed under the control of the endogenous *HMR* **a**1 promoter of each species, in two separate, but otherwise isogenic, hybrid strains (Figure 2A).

**Figure 3:**
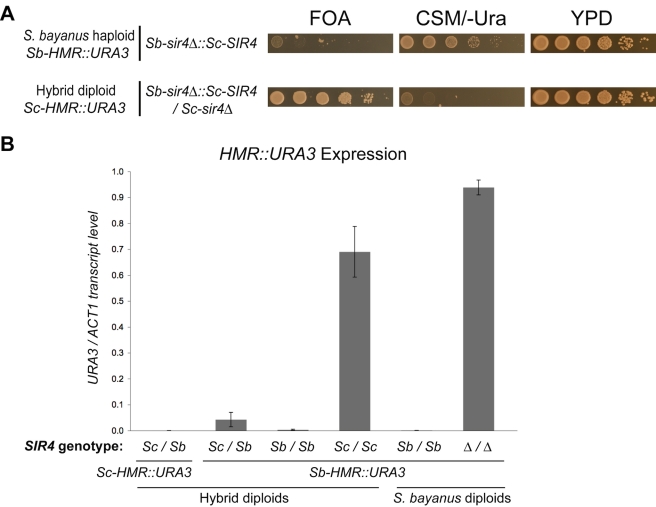
Further characterization of the silencing incompatibility. (A) *Sc-SIR4* was unable to silence *Sb-HMR* in *S. bayanus*. Top row: Silencing of *Sb-HMR::URA3* in an *S. bayanus* haploid strain bearing *Sc-SIR4* integrated in place of *Sb-SIR4*. Bottom row: Control showing that the *Sb-sir4Δ::Sc-SIR4* replacement allele could supply silencing function to *Sc-HMR* in an *S. cerevisiae/S. bayanus* hybrid. (B) RNA analysis of *HMR::URA3* reporters in *S. cerevisiae/S. bayanus* hybrids and *S. bayanus* diploids. *URA3* amplification values were normalized to those of actin (*ACT1*) for each strain. Error bars show standard deviations (*n* = 3).

In these hybrids the *S. cerevisiae SIR4* (*Sc-SIR4*) allele could not, on its own, silence *Sb-HMR* (Figure 2A, row 2). Reduced dosage of Sir4 *per se* did not cause loss of silencing at *Sb-HMR*, as *S. bayanus* diploids with only one copy of *Sb-SIR4* showed no silencing defect (Figure 2B, row 2), nor did *S. cerevisiae* diploids with only one copy of *Sc-SIR4* (unpublished data). Furthermore, a hybrid diploid containing two copies of *Sc-SIR4* (the *Sb-SIR4* gene was replaced by *Sc-SIR4*) also failed to silence *Sb-HMR* (Figure 2A, row 5). In contrast, one *Sc-SIR4* gene was able to silence *Sc-HMR* in all hybrid strains tested (Figure 2A, bottom panel; Figure 3A). Thus, the hybrid cellular environment did not interfere with Sc-Sir4 function, and within a species, *SIR4* was not haplo-insufficient. It appeared that Sc-Sir4 was either inhibited at *Sb-HMR* by something encoded by the *S. bayanus* genome specifically or somehow failed to interact with proteins that promoted *Sb-HMR* silencing.

Transcription analysis of a critical set of the hybrid strains showed good correspondence between expression of the *HMR::URA3* reporter and growth patterns observed on FOA and CSM/-Ura media (Figure 3B).

We note that in the interspecies hybrids with both species' *SIR4* alleles (*Sc-SIR4*/*Sb-SIR4*), *Sb-HMR* silencing appeared weakly defective relative to the complete silencing of *Sb-HMR* in *S. bayanus* diploids by both the reporter assay and direct RNA measurement (compare Figure 2A, row 1 with Figure 2B, row 1; Figure 3B). In addition, *Sb-HMR* silencing was further weakened in hybrids lacking Sc-Sir4 (Figure 2A, compare row 3 with row 1). This result was paradoxical because Sc-Sir4 appeared to have very little ability to silence *Sb-HMR* in hybrids lacking Sb-Sir4. As explained below, these weak *Sb-HMR* silencing defects were likely due to an emergent property of the hybrids, resulting from unusually strong interactions between Sb-Sir4 and *S. cerevisiae* silent loci that effectively reduced Sb-Sir4 associations with *Sb-HMR*. The presence of Sc-Sir4 limited the competition for Sb-Sir4.

### Conditional Association of Sc-Sir4 with *S. bayanus HML* and *HMR*

**Figure 4:**
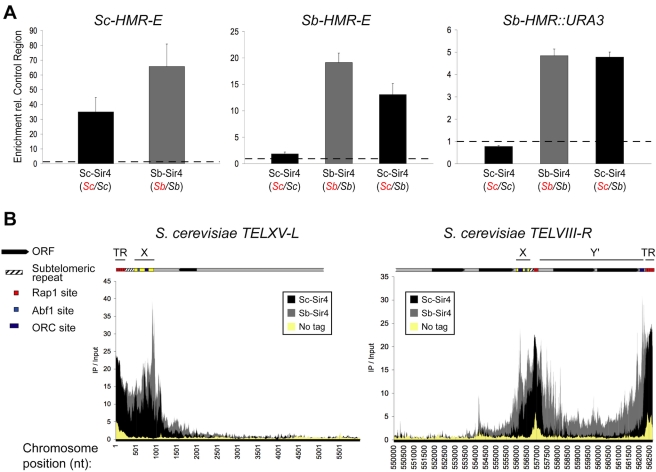
Sc-Sir4 versus Sb-Sir4 ChIP-Seq analysis in *S. cerevisiae/S. bayanus* hybrids. (A) Left: Sir4 IP/Input ratios, normalized to control regions within each experiment, for the *Sc-HMR-E* silencer. Center: Normalized IP/Input ratios for the *Sb-HMR-E* silencer. Right: Normalized IP/Input ratios for the *Sb-HMR::URA3* ORF. “Sc-Sir4” or “Sb-Sir4” labels indicate which species' Sir4 protein was examined by ChIP. Species' identities of both *SIR4* alleles in each strain are given in parentheses, with the allele bearing the 13x-Myc tag indicated in red: (*Sc/Sc*), JRY9062; (*Sb/Sb*), JRY9063; (*Sc/Sb*), JRY9064 (see Table S1 for complete strain genotypes). Dashed lines indicated IP/Input ratio of non-silenced control regions. Error bars indicate the standard error of the mean of all 100 bp windows covering a region. See Table 1 for non-normalized IP/Input ratios and Methods for a description of data processing. (B) ChIP-Seq profiles of Sc-Sir4 (JRY9062), Sb-Sir4 (JRY9063), and the “No tag” control (JRY9054) at two *S. cerevisiae* telomere regions. The ratio of IP/Input read counts for each base of a telomeric region is plotted. Diagrams indicate salient genetic features of two telomeres (see key at left) with X elements (yellow boxes), Y′ elements, and terminal repeats (TR) containing Rap1 binding sites, labeled above. *TELXV-L* (left panel) has an X-element-only end, whereas *TELVIII-R* (right panel) has an X-Y′ end. The *TELVIII-R* Y′ element spans nucleotide positions 556986–562456, with two helicase-encoding ORFs located between positions 558014 and 562047 (www.yeastgenome.org). For the ORFs within this Y′ element, Sc-Sir4 had a mean IP/Input ratio of 1.2, and the “No tag” control had a mean IP/Input ratio of 0.9 (the mean IP/Input ratio for all non-silenced regions, genome wide, was approximately 0.7 for both Sc-Sir4 and Sb-Sir4 ChIPs).

**Figure 5:**
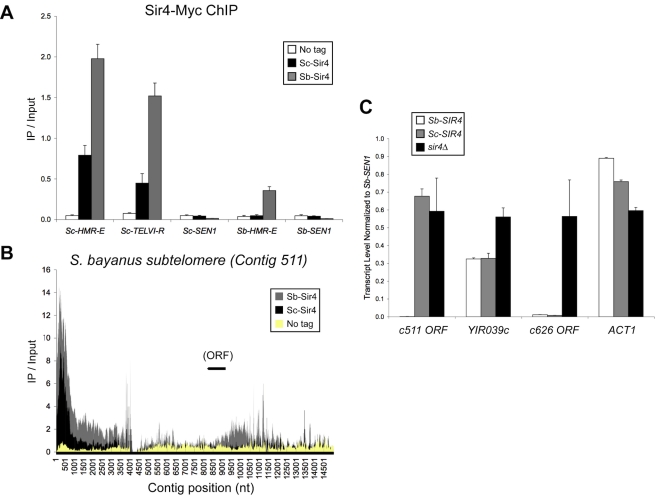
Additional comparative Sir4 ChIP and expression analyses. (A) ChIP-qPCR analysis of Sc-Sir4 versus Sb-Sir4. For each primer set, the IP/Input ratios for Sc-Sir4 (JRY9062), Sb-Sir4 (JRY9063), and the “No-tag” control (JRY9054) are shown. Error bars show standard deviations (*n* = 3). (B) ChIP-Seq analysis of Sc-Sir4 versus Sb-Sir4 association on an *S. bayanus* contig containing subtelomeric sequence (GenBank accession number AACG02000166). Hybrid strains used in this analysis were identical to those used in Figures 4B and 5A: Sc-Sir4, JRY9062; Sb-Sir4, JRY9063; No tag, JRY9054. Per-base IP/Input ratios, determined as in Figure 4B, are plotted versus contig position. We note that the terminal TG_1–3_ repeats are not present in the current *S. bayanus* genome assembly [77]. (C) RNA expression analysis of putative *S. bayanus* subtelomeric genes. Quantitative RT-PCR was performed on RNA isolated from S. bayanus wild-type (*Sb-SIR4*), *Sb-sir4Δ::Sb-SIR4* (*Sc-SIR4*), or *sir4Δ* strains. The actin gene (*ACT1*) served as a euchromatic control gene. Error bars show standard deviations (*n* = 3).

The inability of Sc-Sir4 to function at *Sb-HML* and *Sb-HMR* could have been manifested either during its recruitment or after its assembly into chromatin [35]. To determine where in the assembly of *S. bayanus* silenced chromatin Sc-Sir4 protein was blocked, we compared the ability of Sc-Sir4 and Sb-Sir4 proteins to associate with all silent loci of both species at high resolution using chromatin-immunoprecipitation followed by deep-sequencing of the precipitate (ChIP-Seq). Sir4 ChIP-Seq was performed using hybrid diploids expressing Sc-Sir4 only, Sb-Sir4 only, or both Sc-Sir4 and Sb-Sir4. Because of the sequence divergence between *HML* and *HMR* of the two species, the occupancy of each species' *HML* and *HMR* loci could be evaluated simultaneously. In each strain, only one *SIR4* allele carried a 13xMyc epitope tag [36]. In hybrids expressing Sc-Sir4 only, robust enrichment of *Sc-HML* and *Sc-HMR* silencers was observed as expected, with very weak enrichment of *Sb-HML* and *Sb-HMR* silencers (Figure 4A, Table 1). Strikingly, Sc-Sir4 association with an internal region of *Sb-HMR* was indistinguishable from non-silenced regions. In contrast, as predicted from the genetic results, Sb-Sir4 associated robustly with *HML* and *HMR* loci from both species, and did so most robustly at *S. cerevisiae* silencers (Figure 4A, Table 1). The ChIP-Seq results were validated at *Sc-HMR*, *Sb-HMR*, and control loci using standard ChIP-qPCR analysis (Figure 5A). Thus, Sc-Sir4 showed strongly reduced association with *Sb-HML* and *Sb-HMR* silencers and no detectable association with their internal regions. The relative absence of Sc-Sir4 from these normally silenced regions of the *S. bayanus* genome was consistent with two possibilities. Perhaps Sc-Sir4 could not interact properly with Rap1, ORC, or the *S. bayanus* Sir1 paralogs assembling on their silencers, or perhaps an *S. bayanus* protein was preventing stable association between Sc-Sir4 and *S. bayanus* silencers.

**Table 1:** Average IP/Input ChIP-Seq values for selected regions of the *S. cerevisiae/S. bayanus* hybrid genome.

Region	Sc-Sir4 (Sc*/Sc)	Sb-Sir4 (Sb*/Sb)	Sc-Sir4 (Sc*/Sb)	Sb-Sir4 (Sc/Sb*)
*Sc-HML-E*	12.49	17.99	8.59	10.07
*Sb-HML-E*	2.75	13.78	6.11	8.28
*Sc-HMR-E*	18.58	45.98	12.44	14.51
*Sb-HMR-E*	1.04	7.33	2.85	4.83
*Sc-HMRa1*	1.76	3.33	1.16	1.39
*Sb-HMR::URA3*	0.44	1.86	1.04	1.11
*Sc-Control*	0.54	0.68	0.47	0.40
*Sb-Control*	0.56	0.38	0.22	0.21

Each genetic element indicated at left represents a 600 bp region containing a silencer, an ORF inside an *HMR* locus, or a non-silenced control region. These control regions were located 3 kb to the left of *HMR-E* in either species' genome and correspond to syntenic regions of both species' *YCR095c* genes. Asterisks in column headers indicate which *SIR4* allele bears the 13x-myc tag. Strains used in this analysis, from left to right: JRY9062, JRY9063, JRY9064, JRY9065.

The comparative Sir4 ChIP-Seq data provided a surprising insight into the mechanism of Sir4 incorporation into silent chromatin. Although Sc-Sir4 binding to *Sb-HML* and *Sb-HMR* loci was barely detectable in hybrids expressing Sc-Sir4 only (Figure 4A, center and right panels; Table 1), in hybrids expressing both Sc-Sir4 and Sb-Sir4, Sc-Sir4 binding increased substantially at *Sb-HML* and *Sb-HMR* silencers and internal regions (Figure 4A; Table 1). Thus, despite the poor ability of Sc-Sir4 to associate with *Sb-HML* and *Sb-HMR* on its own, Sb-Sir4 somehow provided Sc-Sir4 access to them. It appeared that Sir4 association with *S. bayanus HML* and *HMR* involved two distinguishable modes of interaction, but Sc-Sir4 was capable of only one (Sb-Sir4-dependent). Moreover, the divergent mode was apparently critical only for the initial association of Sir4 with a silencer, and not for subsequent associations with the silenced region. The ability of Sc-Sir4 to form dimers suggested one straightforward explanation for the Sb-Sir4-dependent chromatin association: inter-specific Sc-Sir4/Sb-Sir4 dimerization through a conserved coiled-coil domain [37],[38].

Sb-Sir4-assisted incorporation of Sc-Sir4 into *Sb-HML* and *Sb-HMR* was consistent with Sc-Sir4 contributing to silencing at these loci, as suggested by the decreased *Sb-HMR* silencing in hybrids lacking Sc-Sir4 (Figure 2A, row 3). However, this hypothesis per se could not explain the sensitivity of *Sb-HMR* silencing to reduced *Sb-SIR4* dosage that was observed in interspecies hybrids, but not in *S. bayanus* diploids (compare Figure 2A, rows 1 and 3, with Figure 2B, row 2; Figure 3B). Further analysis of Sir4 localization on the *S. cerevisiae/S. bayanus* hybrid genome by ChIP-Seq provided an explanation of this hybrid-specific *Sb-SIR4* dosage sensitivity, as described next.

### Differential Association of the Two Sir4 Proteins with Native Telomeric Regions: Sb-Sir4 Sequestration by *S. cerevisae* Subtelomeres

Given the differential association of Sc-Sir4 and Sb-Sir4 with the two species' *HML* and *HMR* loci, we asked if any other loci, genome-wide, also showed a dramatic discrepancy. In *S. cerevisiae*, silencing by Sir proteins occurs at telomeres and subtelomeres, in addition to *HML* and *HMR* [18],[39],[40]. A comparison of the interspecies hybrids expressing Sc-Sir4 only versus Sb-Sir4 only showed that all *S. cerevisiae* TG_1–3_ terminal repeats (which contain embedded Rap1 binding sites), including those present on the centromere-proximal side of some Y′ elements, were comparably occupied by both species' Sir4 proteins (Figure 4B). (Y′ elements are helicase-encoding repetitive sequences of unknown origin and function that are found in some subtelomeric regions immediately adjacent to the terminal repeats [22].) This result was not surprising as the telomerase-replicated repeated sequence, templated by the *TLC1* RNA, is identical in the two species (our unpublished observations). Thus, it appeared that Sir4 association with the *S. cerevisiae* genome, as promoted by Rap1, was not substantially different between Sc-Sir4 and Sb-Sir4. Indeed, the C-terminal residues of Sc-Sir4 critical for its interaction with Rap1 are conserved in Sb-Sir4 (our unpublished observations). We note that the smaller ChIP-Seq peaks observed in these regions in the “No tag” control strain (Figure 4B, yellow shading) are likely due to non-specific DNA binding to the anti-myc beads.

Unexpectedly, *S. cerevisiae* subtelomeres had two types of regions notably more enriched by Sb-Sir4 ChIP than by Sc-Sir4 ChIP. These regions corresponded to X elements, which are regulatory sequences near telomere ends that contain ORC and Abf1 binding sites [22], and the ORFs within Y′ elements. For X elements, ChIP-Seq of Sc-Sir4 showed an average of 7-fold enrichment, whereas Sb-Sir4 showed an average of 14-fold enrichment, with even greater disparity often evident immediately adjacent to X elements (Figure 4B). Therefore, Sb-Sir4 either associated more robustly with factors bound to X elements than did Sc-Sir4 or conceivably was excluded less effectively. X element core sequences (containing the ORC and Abf1 binding sites) are bordered on the telomere-proximal side by X-element combinatorial repeats (formerly known as subtelomeric repeats or STRs; [22]) and the terminal repeats (see http://www.yeastgenome.org/images/yeastendsfigure.html for schematics depicting X-only and X-Y′ telomere ends). The differential pattern of Sir4 association with X elements was consistent with Sb-Sir4 associating more robustly than Sc-Sir4 with sequences at, and immediately adjacent to, the ORC binding sites, presumably via ORC-mediated interactions (Figure 4B). Other *S. bayanus* proteins produced in the hybrids, such as the Sir1 paralogs, may contribute to the enhanced association of Sb-Sir4 with X elements, as discussed below.

We observed weak Sc-Sir4 association with Y′ elements despite its strong association with neighboring terminal repeats (Figure 4B, right panel), consistent with earlier observations using ChIP-chip and transcription reporter analyses [23],[41]. Surprisingly, Sb-Sir4 associated considerably better than Sc-Sir4 with all Y′ elements, showing an average of 5-fold enrichment across their coding regions by Sb-Sir4 ChIP versus 1.2-fold enrichment by Sc-Sir4 ChIP. We note that the *S. bayanus* genome lacks Y′ elements, and thus *S. bayanus* subtelomeres may have reduced Sir4 recruitment potential relative to *S. cerevisiae* subtelomeres [42],[43]. Thus, the enhanced associations of the Sb-Sir4 protein with X and Y′ elements suggested that, in the hybrid strains, *S. cerevisiae* telomeres might have competed with Sb-Sir4 association with *Sb-HML* and *Sb-HMR*, leading to the somewhat weakened *Sb-HMR* silencing observed in hybrids with only one copy of Sb-Sir4 (Figure 2A, rows 1 and 3; Figure 3B). Sb-Sir4 association was indeed reduced at *Sb-HMR* and *Sb-HML* silencers in a hybrid expressing only one copy of *Sb-SIR4*, relative to a hybrid with two copies of *Sb-SIR4* (Table 1, compare columns 2 and 4). Thus, Sc-Sir4 may have, in effect, protected *Sb-HMR* silencing in hybrids when Sb-Sir4 was present (Figure 2A, compare rows 1 and 3) by occupying sites at *S. cerevisiae* telomeres that would otherwise have been bound by Sb-Sir4. (Although the *S. cerevisiae* Y′ elements are bound by Sb-Sir4 and not by Sc-Sir4 in cells with only a single species' Sir4, in the *Sc-SIR4*/*Sb-SIR4* hybrids, Sc-Sir4 and Sb-Sir4 both occupy Y′ elements (unpublished data). However, the extent of occupancy by Sb-Sir4 is less than in cells with Sb-Sir4 only, consistent with Sc-Sir4's ability to spare Sb-Sir4 binding to Y′ elements in the hybrids.)

The ChIP-Seq data allowed us to determine whether the species restriction to Sc-Sir4 association, evident at *Sb-HML* and *HMR*, also applied to *S. bayanus* telomeres. Although subtelomeric regions of the *S. bayanus* genome are presently incompletely assembled and annotated (see *Saccharomyces* Genome Database, www.yeastgenome.org), we identified several candidate subtelomeric contigs based on homology to *S. cerevisiae* subtelomeric genes and X elements. Contigs from the *S. bayanus* genome assembly that contained regions bound by both Sc-Sir4 and Sb-Sir4 (as determined by peak-calling software, see Methods) and putative subtelomeric sequence were further examined for Sir4 ChIP enrichment (an example is shown in Figure 5B). Sb-Sir4 associated with one end of each of these contigs and usually with an internal region as well, typically within 10 kb of the contig end. Interestingly, in the Sc-Sir4-only hybrid, Sc-Sir4 association was observed at the contigs' ends, but not at the internal regions that bound Sb-Sir4. This result suggested that Sc-Sir4, even in the absence of Sb-Sir4, was capable of associating with *S. bayanus* telomere ends, presumably via the conserved Rap1 protein, but could not make some additional contacts necessary to associate with internal sequences.

To test whether the Sc-Sir4 molecules bound to *S. bayanus* telomeres were capable of silencing *S. bayanus* subtelomeric genes, we measured the transcription of candidate subtelomeric ORFs in *S. bayanus* wild-type, *Sb-sir4Δ::Sc-SIR4*, and *Sb-sir4Δ* strains. Importantly, the expression of all three putative subtelomeric genes increased in *Sb-sir4Δ* cells (Figure 5C). Although Sc-Sir4 was capable of silencing *Sb-YIR039c* and an ORF located on Contig_626, it could not repress the transcription of an ORF on Contig_511 located almost 9 kb from the main peak of Sc-Sir4 ChIP. Thus, Sc-Sir4 could bind to and silence at least a subset of *S. bayanus* telomeric regions. It was possible that *S. bayanus* had subtelomeric regulatory elements that promoted silencing, in addition to the Rap1-binding terminal repeats. Depending on the sequence of a particular element, or its proximity to the telomere end, Sc-Sir4 may or may not have been capable of binding and silencing.

### The *Sb-HMR* Silencers Mediated the Species Restriction of Sc-Sir4

**Figure 6:**
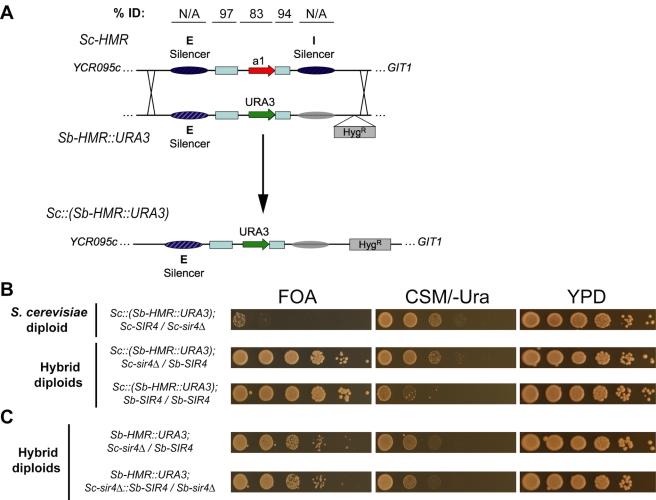
Transfer of *Sb-HMR* into *S. cerevisiae*, identifying *cis*-component of cross-species silencing incompatibility. (A) Schematic diagram depicts replacement of *Sc-HMR* by *Sb-HMR::URA3* in *S. cerevisiae*, creating the *Sc::(Sb-HMR::URA3)* allele. Diagonal lines depict cross-overs for the *HMR* allele swap, with other genetic features of the two *HMR* loci as in Figure 2. A hygromycin-resistance marker (Hyg^R^) was inserted 3 kb to the right of *Sb-HMR* to allow targeted recombination. Shown above is percent identity (BLASTN) between *S. cerevisiae* and *S. bayanus* for the two silencers, the two cassette homology regions (light blue boxes), and the *HMR* **a**1 gene (promoter plus ORF). Note that the silencer sequences show no significant alignment by BLAST. (B) Silencing of the *Sc::(Sb-HMR::URA3)* reporter in *SIR4/sir4Δ S. cerevisiae* diploids (first row), in *Sc-sir4Δ/Sb-SIR4 S. cerevisiae/S. bayanus* hybrids (second row), and in *Sc-sir4Δ::Sb-SIR4/Sb-SIR4 S. cerevisiae/S. bayanus* hybrids (third row). Note that the change in silencing between the first and second rows could be seen only on FOA, with similar growth on CSM/-Ura. This likely reflects *Sb-SIR4* dosage sensitivity, as seen in the original hybrid diploids (Figure 2A). (C) Control strains showing expected silencing functions of *Sc::(Sb-HMR::URA3)* and *Sc-sir4Δ::Sb-SIR4* replacement alleles in interspecies hybrids. Silencing of the *Sb-HMR::URA3* reporter gene, located in its native *S. bayanus* chromosomal context, in *Sc-sir4Δ/Sb-SIR4* hybrids (top row), and in *Sc-sir4Δ::Sb-SIR4/Sb-sir4Δ* hybrids (bottom row). Note that silencing of *Sb-HMR::URA3* in these hybrids was equivalent to *Sc::(Sb-HMR::URA3)* silencing in (B), indicating that the functions of *Sb-HMR* and *Sb-SIR4* were largely unaffected by *S. cerevisiae* chromosomal context.

The cross-species complementation and ChIP analyses suggested that the incompatibility between *Sc-SIR4* and *Sb-HML* and *HMR* was caused by the failure of one or more physical interactions occurring at *S. bayanus* silencers. In principle, the lack of productive Sc-Sir4 association with *Sb-HML* and *Sb-HMR* could have resulted either from an *S. bayanus*-specific inhibitor of silencing that Sc-Sir4 could not overcome or an *S. bayanus*-specific positive regulator of silencing (e.g., Sb-Rap1 or Sb-Sir1) with which Sc-Sir4 could not interact. To distinguish between these models, in an *S. cerevisiae* strain, we replaced the *Sc-HMR* locus with *Sb-HMR* containing the *URA3* reporter, including the flanking silencer elements (Figure 6A). If *S. bayanus* encoded an inhibitor of silencing that Sc-Sir4 could not overcome, *Sb-HMR* should be silenced in *S. cerevisiae*, given the strong conservation of ORC, Rap1, and Abf1 proteins and the Rap1 and Abf1-binding sites in the *HMR-E* silencer [5]. If, however, Sc-Sir4 failed to be recruited to *S. bayanus* silencers, we would expect little or no silencing of *Sb-HMR* in *S. cerevisiae*.

Upon transfer into *S. cerevisiae*, *Sb-HMR* was silenced extremely poorly (Figure 6B, row 1). However, the transplanted *Sb-HMR* locus could still be silenced in the context of the *S. cerevisiae* chromosome in hybrids made by mating the *S. cerevisiae Sb-HMR* strain to wild-type *S. bayanus*. The transplanted *Sb-HMR* locus was silenced to approximately the same degree as the native *Sb-HMR* locus in hybrids (Figure 6B, row 2, compare with 6C rows 1 and 2). The slightly incomplete silencing of the transplanted *Sb-HMR* was largely due to the *Sb-SIR4* dosage sensitivity observed in the original set of hybrids (Figure 2A, row 3), as silencing was strengthened in *Sb-SIR4/Sb-SIR4* hybrids (Figure 6B, row 3). Thus, the lack of silencing of *Sb-HMR* in hybrids expressing only Sc-Sir4 (Figure 2A, rows 2 and 5) was not due to an inhibitor of silencing encoded elsewhere in the *S. bayanus* genome. Rather, the incompatibility was encoded in the *Sb-HMR* locus itself, requiring *S. bayanus*-specific silencing proteins to interpret *Sb-HMR*-specific sequence information. These “interpreter” proteins potentially included DNA-binding proteins such as ORC, Rap1, and Abf1 or proteins indirectly associated with silencers, such as Sir1, Sir4, or both.

**Figure 7:**
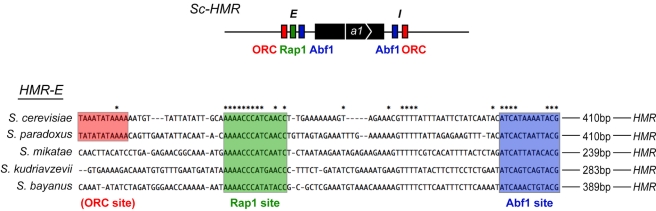
Structure and conservation of binding sites in the *HMR-E* silencer in the *sensu stricto* clade. A multiple alignment of five species' *HMR-E* sequences was produced by ClustalW. Note the strong conservation of the Rap1 and Abf1 binding sites (all 7 bp of the Abf1 consensus site were conserved), with poor conservation of the ORC binding site and intervening sequences. Asterisks indicate a nucleotide is conserved across all species. Adopted and modified from [5].

Alignments of *Sc-HMR* and *Sb-HMR* suggested that their functional divergence was due to changes in the silencer sequences between the two species (Figure 7). The *HMR* **a**1 gene was 83% identical between *S. cerevisiae* and *S. bayanus* (the promoter was 93% identical), well above the genome-wide average of 62% identity for all intergenic regions, and the mating-type cassette-homology sequences (shared with *MAT* and *HML*) approached 100% identity (Figures 6A and 7). Notably, the silencer sequences share well below the genome-wide average identity for intergenic regions and are difficult to align outside of the conserved Rap1 and Abf1 sites [5].

### Reconstitution of *S. bayanus* Silencing in *S. cerevisiae* with *Sb-SIR4* and *Sb-KOS3*

**Figure 8:**
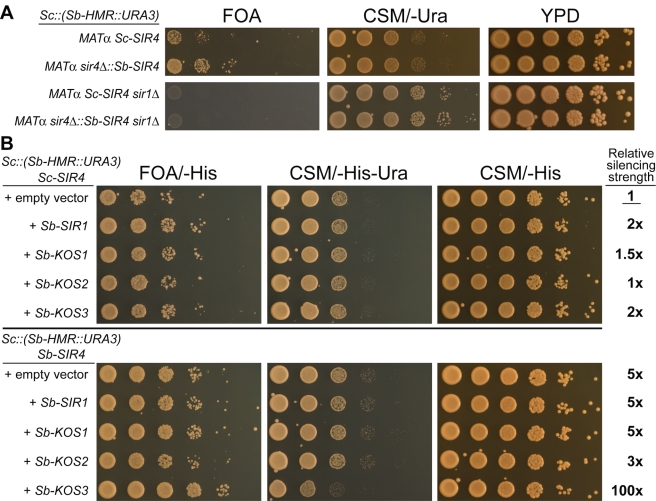
Partial reconstitution of *Sb-HMR* silencing in *S. cerevisiae* by transfer of *S. bayanus* Sir4 and Kos3 proteins. (A) Top panel: Silencing of the *Sc::(Sb-HMR::URA3)* replacement allele in *S. cerevisiae MAT*α haploids bearing either the endogenous *Sc-SIR4* gene or an integrated *Sb-SIR4* gene (top panel). Bottom panel: Silencing of the *Sc::(Sb-HMR::URA3)* replacement allele in the absence of *Sc-SIR1*. (B) *S. cerevisiae* strains bearing the *Sc::(Sb-HMR::URA3)* replacement allele, and either the endogenous *Sc-SIR4* gene or an integrated *Sb-SIR4* gene, were transformed with plasmids encoding individual *S. bayanus* Sir1 paralogs and assayed for silencing function (FOA/-His, CSM/-His-Ura, or CSM/-His indicate silencing reporter media also selective for maintenance of plasmids bearing the *HIS3* marker). Quantification of relative silencing function, based on growth on FOA/-His, is indicated at right. Fold-change comparisons were made relative to the *Sc::(Sb-HMR::URA3) Sc-SIR4* strain bearing an empty vector (row 1). We note that the *CEN/ARS* plasmid itself appeared to enhance *Sb-HMR* silencing relative to the untransformed strains (compare Figure 8B, “empty vector” rows, to Figure 8A, rows 1 and 2). However, relative comparisons among transformed strains were still possible.

The simplest model consistent with the results so far was that the silencing incompatibility was limited to Sir4, with Sc-Sir4 having a more restricted range of interactions than Sb-Sir4. To test this possibility, we replaced *Sc-SIR4* with *Sb-SIR4* in the *S. cerevisiae* strain bearing *Sb-HMR*. If the incompatibility involved only *SIR4* and silencers, *Sb-SIR4* should restore silencing to *Sb-HMR*. Indeed, the *S. cerevisiae* strain with *Sb-SIR4* and *Sb-HMR* indeed showed a modest increase in silencing relative to the *Sc-SIR4 Sb-HMR* strain, confirming that changes in Sir4 itself contributed to the silencing incompatibility. However, this silencing increase—a 5-fold change—was detectable only as an increase in FOA resistance, and was still at least 100-fold below the level of *HMR* silencing seen in the hybrids (Figure 8A, row 2; compare with Figure 6B, row 2). Thus, although a portion of the incompatibility could be explained by *SIR4* and silencer co-evolution, one or more additional *S. bayanus* proteins were likely required to recruit Sb-Sir4 efficiently or to stabilize its association with *S. bayanus* silencers.

Interestingly, Sc-Sir4's very weak ability to silence the transplanted *Sb-HMR* locus resulted in the low-frequency appearance of FOA-resistant colonies (occurring at an approximate frequency of 5×10^−5^; Figure 8A, row 1). Within these colonies, which grew at nearly the rate expected of Ura- strains, the cells were able to grow under conditions that killed the majority of cells that did not form colonies. Hence this silencing occurred at low frequency, but was nonetheless heritable. Indeed, *Sb-HMR* silencing by either Sb-Sir4 or Sc-Sir4 was fully dependent on *S. cerevisiae* Sir1 (Figure 8A), whose role is to promote the establishment of heritable silencing. That *Sb-HMR* could be silenced at all in *S. cerevisiae* suggested that a critical subset of Sc-ORC, Rap1, and Abf1 bound productively to *Sb-HMR* silencers. It was therefore possible that providing additional *S. bayanus* silencing proteins could stabilize interactions between the *S. cerevisiae* DNA-binding proteins and *S. bayanus* silencers. Likely candidates to provide this presumptive function were the *S. bayanus* Sir1 paralogs, Kos1, Kos2, and Kos3, with Kos3 being the most structurally distinct from Sir1, yet the most similar to the ancestral member of the Sir1 family [24]. Interestingly, *Sb-KOS3* enhanced *Sb-HMR* silencing synergistically with *Sb-SIR4*, but not with *Sc-SIR4* (Figure 8B; compare rows 1, 5, and 10). None of the other Sir1 paralogs of *S. bayanus* provided a dramatic enhancement of *Sb-HMR* silencing. The *Sb-HMR Sb-SIR4 +Sb-KOS3* strain showed 100-fold better silencing than the *Sb-HMR Sc-SIR4* strain (Figure 8B, compare rows 1 and 10). This result was particularly interesting because Sir4 interacts weakly and non-specifically with DNA [44], and Kos3 is not thought to bind DNA at all. Thus, the “interpretation” of differences between the *Sb-HMR* and *Sc-HMR* silencers by Sb-Kos3 and Sb-Sir4 presumably required some sort of *HMR*-allele-specific collaboration with silencer-binding proteins that could be interpreted by Sb-Kos3 and Sb-Sir4 in a species-specific way.

### Differential ORC Utilization by *S. bayanus* Silencers

**Figure 9:**
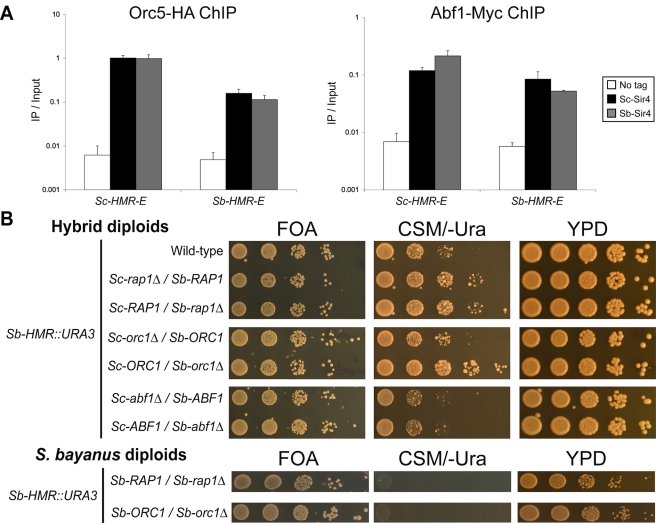
ChIP and genetic interaction analysis of ORC, Rap1, and Abf1 silencing functions. (A) ChIP analysis of Sc-Orc5 and Sc-Abf1 in *S. cerevisiae* at *Sc-HMR-E* versus *Sb-HMR-E*. Relative enrichment of silencer sequences was verified by comparison to amplification values for a positive control region, the *ARS1* replication origin, and a negative control region in the *SEN1* gene (unpublished data). Note log scale on *y*-axis. Error bars show standard deviations (*n* = 3). (B) Top panel: Silencing of the *Sb-HMR::URA3* reporter gene in *S. cerevisiae/S. bayanus* hybrids each lacking a single allele of the *RAP1*, *ORC1*, or *ABF1* genes. Bottom panel: Silencing of the *Sb-HMR::URA3* reporter gene in *S. bayanus* diploids lacking one allele of *RAP1* or *ORC1*. Note that to properly document the silencing differences shown in (B), the CSM/-Ura plates were photographed after 5 d, whereas in Figure 2 CSM/-Ura plates were photographed after 3 d.

By sequence conservation, Rap1 and Abf1 binding sites can be detected in the *Sb-HMR-E* silencer, but the ORC binding site is not readily identified (Figure 7) [5]. Given Sb-Sir4's dependence on Sir1 and Kos3, and their dependence on ORC [20],[45],[46], our results suggested two likely explanations for why *Sb-HMR* was not silenced in *S. cerevisiae*: either Sc-ORC bound *S. bayanus* silencers less well than *S. cerevisiae* silencers, or Sc-ORC bound equivalently but failed to promote silencing because it was in a suboptimal conformation or context with respect to other silencer binding proteins. In either case, the subsequent interactions with Sc-Sir1 and Sc-Sir4 might suffer. To test whether Sc-ORC indeed bound to *S. bayanus* silencers, we performed ChIP analysis on HA-tagged Sc-Orc5 in *S. cerevisiae* bearing *Sb-HMR*. Sc-Orc5 associated with the *Sb-HMR-E* silencer, albeit at a level several-fold below its association with *Sc-HMR-E* (Figure 9A, left panel; note log scale on *y*-axis). A parallel analysis with Sc-Abf1 ChIP showed robust association of this protein with both *Sc-HMR-E* and *Sb-HMR-E* silencers (Figure 9A, right panel). We note that both Sc-Orc5 and Sc-Abf1 associations with *Sb-HMR-E* showed small alterations in the *Sb-SIR4* strain relative to the *Sc-SIR4* strain. However, these changes did not correlate with *Sb-HMR* silencing levels (Figure 8A, rows 1 and 2). These ChIP data were consistent with *Sb-HMR* silencers having conserved functional binding sites for ORC and Abf1.

To test whether Sc-ORC, Rap1, and Abf1 indeed participated in *S. bayanus* silencing, we monitored silencing of *Sb-HMR* in hybrids lacking either species' complement of each of these proteins (out of the six ORC subunits, we focused on Orc1 because it directly interacts with Sir1). Because *RAP1*, *ABF1*, and *ORC1* are essential, we assayed silencing in hybrids heterozygous for each gene. *S. cerevisiae* diploids sensitized to detect silencing defects at *HMR* show strong silencing defects if either *SIR1* or *SIR4* dosage is also reduced [47]. Similarly, *Sb-HMR* silencing was weakly compromised in hybrids whereas *Sc-HMR* was not (Figure 2A), potentially providing a sensitized background to uncover similar types of genetic interactions. For this reason, any such silencing defects in heterozygous hybrids were expected to affect silencing of *Sb-HMR* but not *Sc-HMR*. Indeed, *Sb-HMR*, but not *Sc-HMR*, was further derepressed in hybrids lacking either *Sc-RAP1* or *Sb-RAP1* (Figure 9B, top panel; Figure S3). Note that *Sb-HMR* was fully silenced in *S. bayanus RAP1/rap1Δ* diploids; therefore, reduced *RAP1* dosage per se did not cause the loss of silencing observed in the hybrid (Figure 9B, bottom panel). Thus, Sc-Rap1 participated in *Sb-HMR* silencing in hybrids, likely by direct binding to *S. bayanus* silencers. In contrast to the analysis with *RAP1*, *Sb-HMR* was derepressed to a greater extent in hybrids lacking *Sb-ORC1* but not in hybrids lacking *Sc-ORC1* (Figure 9B, top panel). Again, *Sb-HMR* was fully silenced in *S. bayanus ORC1/orc1Δ* diploids (Figure 9B, bottom panel), ruling out simple dosage explanations. Hence, Sb-Orc1 was more important for *Sb-HMR* silencing in hybrids than Sc-Orc1, suggesting that a partial species restriction existed with respect to ORC binding or activity at *Sb-HMR* silencers. Heterozygosity of *ABF1* had no effect on either *Sb-HMR* or *Sc-HMR* silencing (Figure 9B, top panel; Figure S3).

### Evidence for Positive Selection on *SIR4*

The rapid sequence and functional divergence of *SIR4* between closely related species suggested that an interesting evolutionary force may have contributed to the functional divergence of this gene. To test whether a specific function of the Sir4 protein had been under positive selection within the *sensu stricto* clade, we aligned *SIR4* coding sequence from all five species and computed the ratio of nonsynonymous to synonymous divergence (henceforth ω, also known as dN/dS) across the whole gene. The value of ω for *SIR4* was 0.44, substantially higher than the genomic average of 0.10. Only 16 of 4,894 loci we analyzed had a higher ω, indicating that *SIR4* was indeed one of the most rapidly evolving genes in the budding yeast genome.

**Figure 10:**
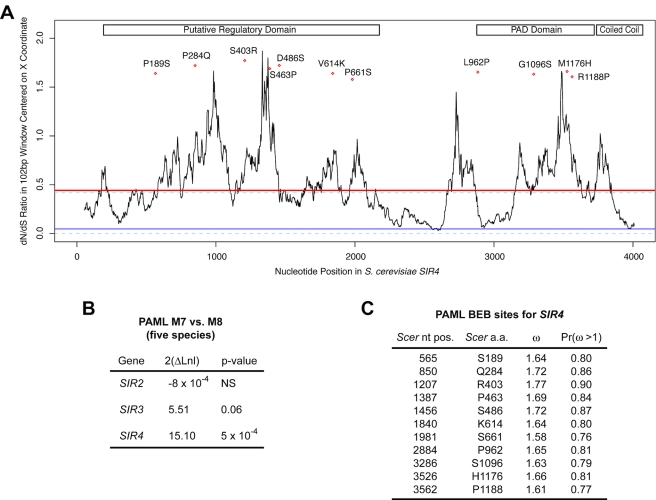
Evolutionary analyses of *SIR4* in the *sensu stricto* clade. (A) Ratios of nonsynonymous to synonymous divergence (dN/dS, or ω) computed in 102 bp windows every 3 bp along alignments of the *SIR4* gene from five *sensu stricto* species (*S. cerevisiae*, *S. paradoxus*, *S. mikatae*, *S. kudriavzevii*, and *S. bayanus*). Horizontal lines show the value of ω for the median *SIR4* window (red solid line), the median window of all genes (blue solid line), and the limits within which 95% of all ∼1,500 windows, sampled from across coding regions of the genome, fall (dashed lines). Diamonds indicate codons having a posterior probability of (ω>1)≥0.75 (corresponding to positions given in panel C). Each of these 11 rapidly evolving codons is labeled with the inferred ancestral amino acid at that position, the amino acid number, and the amino acid present in *S. cerevisiae* Sir4. Labeled boxes at top indicate the locations of functional domains. (B) Table summarizing statistics of PAML's M7 versus M8 evolutionary models for *SIR2*, *SIR3*, and *SIR4*. For each gene, the starting nucleotide alignments were generated using sequences from the five *sensu stricto* species used in panel A (see Methods for further description of PAML analysis). NS, not significant. (C) Table summarizing a subset of the Bayes Empirical Bayes (BEB) analysis for model M8 of PAML. The identities of the 11 sites with posterior probability of (ω>1)≥0.75 are shown. Nucleotide (nt) and amino acid (a.a.) positions from *S. cerevisiae SIR4* are given.

A value of ω significantly greater than 1 is evidence of positive selection [48]. Therefore, a value of 0.44 might suggest that the *SIR4* coding region did not evolve under positive selection. However, because Sir4 is a large protein we investigated whether sub-regions or individual codons might have ω>1. To determine whether rapidly evolving Sir4 residues might lie within known functional regions of the protein, we computed ω in 102 bp (34-codon) windows throughout the *SIR4* open reading frame (Figure 10A). Consistent with our previous whole-gene estimate, the median ω value for all windows in *SIR4* was 0.43 (Figure 10A, solid horizontal line) with a range from 0.02 to 1.87. Because ω estimates calculated in short windows are subject to stochastic noise, we compared the results of this analysis to ∼1,500, 102 bp windows drawn from other *S. cerevisiae* coding regions. The median of these ω values was 0.05, and 95% of windows lie between 0.0001 and 0.42 (Figure 10A, dashed lines). These comparisons supported two conclusions. First, because the median ω for *SIR4* was comparable to the most extreme values in other genes, the unusual molecular evolution of this gene extended over a large fraction of its length. Second, the non-random distribution of windows with high ω suggested that the rapid evolution of certain residues was connected to functional changes within specific regions of the Sir4 protein. In support of this suggestion, simulations of *SIR4* evolution indicated that 1.3% of 102 bp windows are expected to have ω>1 by chance compared to 6.7% observed in *sensu stricto SIR4* (unpublished data). The high-ω windows in *SIR4* were therefore unlikely to reflect noise and instead indicated that the most rapidly evolving codons are concentrated in particular regions of *SIR4*. Indeed, although the Rap1- and Sir3-binding coiled-coil domain was largely protected from the rapid evolution of *SIR4*, residues within the PAD (Partioning and Anchoring of plasmids) and the putative N-terminal regulatory domains [37] showed striking signatures of rapid evolution (Figure 10A).

To provide an independent, statistically robust analysis of *SIR4* evolution in this clade, we used a likelihood-ratio test to compare nested models of sequence evolution that either allowed or did not allow a subset of codons to have a value of ω>1. The model allowing ω>1 (M8) fit the data significantly better than the alternative model (M7; *p* = 5×10^−4^), indicating that some codons were likely to be evolving under positive selection (Figure 10B). The posterior probability of ω≥1 exceeded 0.75 for 11 codons (Figure 10A and C; ω≥1.5 in all cases), however for no single codon did the posterior probability exceed the nominal significance level of 0.95. Inclusion of *SIR4* sequences from species outside the *sensu stricto* was not possible because of poor alignment quality. In summary, although we were not able to identify specific codons that were unambiguously under positive selection, these data suggested that multiple codons within *SIR4*, including some within the PAD and N-terminal regulatory domains, exhibit signatures of extremely rapid sequence evolution in the *Saccharomyces sensu stricto* clade.

To examine whether the rapid sequence evolution of *SIR4* showed a phylogenetic correlation with the functional divergence we observed, we fit models that allowed different branches of the *SIR4* tree to have different values of ω. If such a correlation were observed (i.e., more rapid evolution of *SIR4* along the *S. cerevisiae* lineage than along the *S. bayanus* lineage), then positive selection on a specific silencing function of *SIR4* during the evolution of *S. cerevisiae* would be likely. Although increased estimates of ω were obtained for some branches (notably the shared *S. cerevisiae/S. paradoxus* branch; ω = 0.55), none were statistically supported, suggesting that there have been no dramatic shifts in the selection pressures operating on *SIR4* since the divergence of the *sensu stricto* (Figure S4). We note that a change in selection pressure that affected only a subset of codons could easily have gone undetected.

## Discussion

Using interspecies hybrids, we have shown by three functional criteria—cross-species complementation assays, cross-species *cis-trans* tests, and genome-wide localization by ChIP-Seq—that the functions of both the Sir4 protein and multiple silencer elements have strikingly diverged over the short divergence time between closely related yeast species. Cross-species complementation assays revealed an incompatibility between Sc-Sir4 and *Sb-HML* and *Sb-HMR* (Figure 2A). The inability of Sc-Sir4 to silence *Sb-HML* and *Sb-HMR* was due to a difference in the protein sequence of Sir4 between the two species rather than a difference in expression level (Figure 10, Figure S2). This incompatibility likely resulted from the coordinated divergence of multiple heterochromatin determinants: Sir1, Sir4, and silencers. Two pieces of evidence implicated *cis*-acting changes in silencer sequences as being key to the incompatibility. First, comparative ChIP-Seq analysis of Sir4 pinpointed an inability of Sc-Sir4 to associate stably with *S. bayanus* silencers (Figure 4A, Table 1). Second, and more definitively, transfer of the *Sb-HMR* locus into *S. cerevisiae* demonstrated that this locus was inherently unrecognizable to Sc-Sir4 (Figure 6B). This result established that *S. bayanus* did not produce an inhibitor of Sc-Sir4 function, and mapped the locus of the Sir4 species specificity to the *Sb-HMR* silencers. As silencing of the transplanted *Sb-HMR* locus was largely restored in an *S. cerevisiae/S. bayanus* hybrid (Figure 6B), *S. bayanus*-specific proteins were required to assemble silent chromatin at *Sb-HMR* in the manner dictated by the *Sb-HMR* silencers, with Sb-Sir4 and the Sb-Sir1 paralogs being the most likely candidates for species-specific “interpreter” proteins.

### Co-Evolution of Silencer Elements and Heterochromatin Proteins in Budding Yeast

The Sir4 protein and silencers diverged rapidly in concert, a process that was accompanied by loss of three Sir1 paralogs in the *S. cerevisiae* lineage [24]. As silencing was robustly maintained in each species, it was likely that these factors had co-evolved such that coding changes in Sir4 and a reduction in Sir1 family members led to compensatory changes in silencers, or vice versa. The asymmetrical complementation of *SIR4* alleles (Figure 2A), and the enhanced ability of Sb-Sir4 to bind *S. cerevisiae* silent loci compared to its own silent loci (Figure 4B), suggested that *S. cerevisiae* silencer elements had become stronger than those of *S. bayanus*, while *S. cerevisiae* Sir1 and Sir4 proteins had become weaker (operationally defined) than *S. bayanus* Sir4 and its four Sir1 paralogs. The intra-species combinations of Sir1 and Sir4 proteins and silencers allowed efficient nucleation of silencing complexes at *HML* and *HMR* in each species.

Broadly speaking, we imagine two possible evolutionary paths for this co-evolution, with variations on either path possible. In an “adaptive” model, hypothetical selective pressure(s) induced coding changes in Sir4 and reduction in Sir1 family members (Zill et al. in preparation), which then required “strengthening” mutations (for example, a change that increased the affinity of ORC for a silencer) in the silencers to maintain robust silencing. In a “constructive neutral” model [49], strengthening mutations accumulated in silencers at random, thus relaxing the selective constraints to maintain Sir1 paralogs and certain Sir4 residues. Once Sir1 paralogs were lost, the “stronger” silencers would need to be maintained by purifying selection. Our evolutionary analyses using the PAML software supported a role for positive selection acting on multiple sites within *SIR4* during the evolution of the *sensu stricto* species (Figure 10). However, the *SIR4* gene showed an unusually high evolutionary rate across most of its length. Furthermore, although we identified 11 rapidly evolving residues that may suggest specific regions' contributions to the functional divergence of Sir4, none of these crossed the 0.95 threshold for statistical significance. (We note that 4 of the 11 fastest evolving sites were localized within the PAD domain, which mediates interactions between Sir4 and Esc1 at the nuclear periphery, and between Sir4 and the Ty5 retrotransposon integrase protein. Interestingly, Esc1 is also one of the most rapidly diverging proteins in *Saccharomyces* species [O. Zill, unpublished observations].) Extensive directed mutational analyses will be necessary to test whether the sites under selection are responsible for the functional divergence between Sc-Sir4 and Sb-Sir4.

An important question relevant to these models is, in which lineage did the observed changes in Sir4 and silencer function occur relative to the common ancestor of *S. cerevisiae* and *S. bayanus*? Although accurate determination of the ancestral state of the silencing mechanism will require extensive evolutionary analyses, it appears that *S. bayanus* has retained at least two ancestral characters that *S. cerevisiae* has lost. First, Kos3, the ancestral Sir1-related protein, has been lost in *S. cerevisiae*. Second, the *SIR4* gene from *K. lactis*, an outgroup to the *Saccharomyces* clade, was able to complement silencing function in *S. bayanus sir4Δ* mutants (Zill et al. in preparation). That a Sir4 protein from a species outside of *Saccharomyces* is compatible with *S. bayanus* silencers suggests that these elements did not “gain” a restrictive property in the *S. bayanus* lineage. The more likely scenario is that Sir4 changed in the *S. cerevisiae* lineage such that its range of interactions with other species' silencers has become restricted, consistent with earlier observations of cross-species function of Sir4 [50]. It will therefore be of interest to understand in detail the mechanism of silencing in *S. bayanus* and to determine what forces caused the dramatic shift in Sir1 and Sir4 functionality in the *S. cerevisiae* lineage. We measured the rates of *SIR4* evolution (ω) along all branches in the *sensu stricto* clade but did not observe a notable asymmetry in these rates (Figure S4). Thus, the functional asymmetry between Sc-Sir4 and Sb-Sir4 was probably localized to a few sites and may not be related to the broad evolutionary forces that have acted on *SIR4* across all five species in this clade.

**Figure 11:**
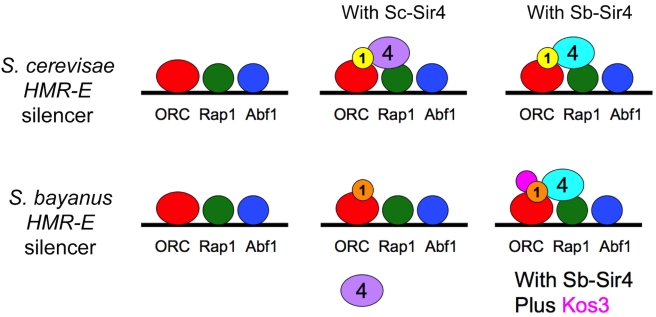
A new twist on co-evolution of transcriptional regulatory proteins and DNA target sites. Although the site-specific DNA binding proteins, ORC, Rap1, and Abf1 were largely interchangeable between *S. cerevisiae* and *S. bayanus*, the Sc-Sir4 protein showed a striking inability to function on *S. bayanus* silencers. *Cis*-regulatory information in the *S. bayanus* silencer was specifically tuned to the Sb-Sir4 and Sb-Kos3 silencing proteins, as shown by reconstitution experiments in *S. cerevisiae* (Figure 8). Loss of Kos3 and changes in Sir4 in the *S. cerevisiae* lineage were compensated by changes in the silencers, thereby maintaining robust silencing.

Perhaps the most striking finding of this study was that the heterochromatin proteins that showed the most dramatic evidence of co-evolution with silencers, Sir1 and Sir4, were not the ones that bind specific DNA sites. Rather, these proteins associate with DNA indirectly via the conserved regulatory proteins Rap1, Abf1, and ORC (Figure 11). The key evidence demonstrating functional co-evolution between Sir4 and the Sir1 family and silencers came from attempts to reconstitute *Sb-HMR* silencing in *S. cerevisiae*. The changes in Sir4 sequence were not sufficient to explain the inability of Sc-Sir4 to function at *S. bayanus* silencers: expression of Sb-Sir4 in an *S. cerevisiae* strain was only modestly effective in silencing an *Sb-HMR* locus transplanted into that strain (Figure 8A). The Sir1-dependence of the rare, but heritable, silencing events mediated by Sb-Sir4 at *Sb-HMR* in *S. cerevisiae* suggested that the limitation involved proteins dedicated to establishing silencing. Indeed, adding Sb-Kos3, the ancestral member of the Sir1 family, together with Sb-Sir4 enhanced silencing of *Sb-HMR* in *S. cerevisiae* by 100-fold (Figure 8B), although not completely. It was possible that the site-specific DNA-binding proteins ORC, Rap1, and Abf1 had also co-evolved with silencer sequences. If this were the case, we would expect hybrids lacking the Sb-ORC, Sb-Rap1, or Sb-Abf1 proteins to have shown defective *Sb-HMR* silencing. However, only Sb-Orc1 inactivation (and by inference, inactivation of the entire Sb-ORC complex) showed the expected *S. bayanus* allele-specific effect on *Sb-HMR* silencing (Figure 9B). This effect of *Sb-ORC1* deletion on *Sb-HMR* silencing was relatively modest, and the addition of *Sb-ORC1* (together with *Sb-SIR4*) had no effect on *Sb-HMR* silencing in *S. cerevisiae* reconstitution experiments (unpublished data). Because Sc-Orc1, Sc-Rap1, and Sc-Abf1 were capable of supporting *Sb-HMR* silencing in hybrids (Figure 9B) and in *S. cerevisiae* (Figure 8B), their DNA-binding domains' interactions with silencers were largely conserved across species and hence were not engaged in notable co-evolution with silencers or with Sir4. Indeed, we were able to ChIP Sc-Orc5 and Sc-Abf1 on the *Sb-HMR-E* silencer in *S. cerevisiae* (Figure 9A). Together, these results suggested that the *cis*-acting differences between the two species' silencers were interpreted largely indirectly, via interactions between ORC, Sir1/Kos3, and Sir4, with a somewhat lesser contribution of differences in ORC-silencer DNA interactions.

### Asymmetrical Interactions of Heterochromatin Determinants in Interspecies Hybrids Yielded Insights into the Silencing Mechanism

Why did Sc-Sir4 not bind efficiently to *S. bayanus* silencers? Simple explanations such as sequence divergence between *S. cerevisiae* and *S. bayanus* silencers precluding sequence-specific contacts with Sir4 are unlikely because biochemical data on Sir4 point to a lack of sequence-specific binding to DNA [44]. Instead, Sir4 is recruited to silencers predominantly via protein-protein interactions [20],[37],[51]. It is unlikely that different proteins bind the silencers in the two species as the preponderance of evidence points to ORC, Rap1, and Abf1 as the critical silencer-binding proteins in both species (Figures 5, 7, and 9). Further, the residues mediating Sc-Orc1 interaction with Sc-Sir1 [52],[53] are conserved in Sb-Orc1 (our unpublished observations). Hence we are forced to consider models in which something special about how ORC, Rap1, and Abf1 bind *S. bayanus* silencers prevents Sc-Sir4 from interacting with Rap1 or creates a requirement for specific interactions with the Sir1 paralogs that can only be made by Sb-Sir4. Perhaps the precise juxtaposition or conformation of these site-specific DNA-binding proteins allow or restrict interactions with a particular species of Sir4. Alternatively, perhaps a reduced affinity of *S. bayanus* silencers for ORC or Rap1, or the ensemble of nucleation proteins, is compensated by binding energy provided by Sb-Kos3 (and possibly additional Sir1 paralogs) and Sb-Sir4, but not by Sc-Sir4. Indeed, complete silencing of both *Sb-HML* and *Sb-HMR* requires Sb-Sir1, Sb-Kos1, and Sb-Kos2 [24]. Therefore, it is possible that a relatively weak binding site (such as for Rap1) in the *Sb-HMR-E* silencer could be compensated by increased binding energy provided to the nucleation complex in *trans* by the combination of Sb-Sir4 and the Sir1 paralogs. Additionally, we note that a requirement for multivalent interactions may help explain why Sir4 fails to interact stably with Rap1 at the many Rap1 binding sites throughout the genome.

An unexpected finding of the Sir4 comparative ChIP-Seq experiment provided insight into the mechanism of silent chromatin assembly. The Sb-Sir4-assisted Sc-Sir4 incorporation into *Sb-HML* and *HMR* (Figure 4A) suggested two distinct types of interactions made by Sir4 proteins at these loci: only Sb-Sir4 was capable of making stable contacts either with the Sir1 paralogs, or perhaps with Rap1. However, as Sc-Sir4 was capable of mediating telomeric silencing at some *S. bayanus* telomeres (Figure 5C), it appeared that Sc-Sir4 could interact productively with the Sb-Rap1 protein. In addition, there was a second and qualitatively distinct mode of Sir4 protein association that was species-independent but occurred only if the species-specific interaction occurred. Three types of interactions might account for the secondary mode of Sc-Sir4 association with *Sb-HML* and *Sb-HMR*: direct Sb-Sir4-Sc-Sir4 interaction via a conserved dimerization surface [38], Sc-Sir4 binding to Rap1 via the conserved C-terminal coiled-coil domain, or Sc-Sir4 interaction with deacetylated histone tails [18]. We note that Sc-Sir4 association with the *Sb-HMR-E* silencer increased in the presence of Sb-Sir4 at least as much as did its association with internal regions of *Sb-HMR* (Figure 4A). Thus, this secondary mode of Sir4 interaction did not appear to be restricted to regions of *Sb-HMR* where the deacetylated histones reside (silencers are nucleosome-free regions). Further studies will resolve whether Sb-Sir4-assisted Sc-Sir4 incorporation involves contacts with multiple silencing proteins versus simple Sir4-Sir4 dimerization and whether it requires Sir2 catalytic activity.

Additionally, the enhanced interaction of Sb-Sir4 across Y′ elements at *S. cerevisiae* telomeres (Figure 4B) suggested that novel or changed interactions in the hybrids somehow led to enhanced Sir4 occupancy of these regions. This differential long-range occupancy by Sir complexes presents an opportunity to ask whether Sir1 and Sir4-mediated interactions during Sir complex nucleation regulate the “strength” of silent chromatin over a distance. Alternatively, Sb-Sir4 (and potentially other *S. bayanus* silencing proteins) may have been less sensitive to factors that exclude Sc-Sir4 from the Y′ elements. The species-specific Sir4 distributions occurring in these interspecies hybrids should be further dissected to understand the determinants limiting silent chromatin formation across subtelomeric regions.

Another unusual property of the interspecies hybrids led to a weak silencing defect affecting *Sb-HMR* but not *Sc-HMR* (Figure 2A, row 1; Figure 3B). In hybrids lacking Sc-Sir4 this defect was more evident (Figure 2A, row 3), which paradoxically suggested that Sc-Sir4 protected *Sb-HMR* silencing in the presence of Sb-Sir4, despite having no ability to silence *Sb-HMR* on its own. How might Sc-Sir4 have “enhanced” Sb-Sir4 function at *S. bayanus* silent loci in hybrids? Strong evidence compatible with Sc-Sir4 protecting Sb-Sir4 from being titrated by Sc-specific sequences was the ability of both Sb-Sir4 and Sc-Sir4 to bind extensively to *S. cerevisiae* telomeres, as described in the Results. Hence, the hybrid state may result in a dosage sensitivity to *Sb-SIR4* not evident in *S. bayanus SIR4/sir4Δ* intra-species diploids due to additional binding sites provided by the Sc-X and Y′ elements, and potentially other elements. We note the resemblance of this “Sb-Sir4 sequestration” model to the “Circe effect” proposed to explain Sc-Sir4-mediated clustering of *S. cerevisiae* telomeres [54].

### On the Special Properties of Interspecies Hybrids with Regard to Heterochromatin

Gregor Mendel's studies were motivated by a desire to understand the emergent properties of interspecies hybrids, such as hybrid vigor, that were of great practical significance at the time. Although he became famously distracted by discovering two fundamental laws of genetics, his original interest in the processes by which hybrid species are not necessarily the “average” of the two parental species remains as interesting today as it was practically important in Mendel's day. Indeed, the striking asymmetry in the ability of Sb-Sir4 to silence *Sc-HMR*, but inability of Sc-Sir4 to silence *Sb-HMR* (Figure 2A), was the seminal observation that inspired this study. By and large, however, in interspecies hybrids of *S. cerevisiae* and *S. bayanus*, a protein from either species was fully capable of providing all of that protein's function to hybrids. Although this result could be anticipated from the ability to “clone by complementation” genes of one species by their function in another, this study established that symmetry of complementation is an important general consideration. For example, the essential proteins Rap1 and Abf1 from either species had all the functions necessary to support viability of the hybrids, and we established that Sir2 and Sir3 of both species were fully interchangeable (Figure S1), despite being members of a complex in which another member of that complex, Sir4, has extraordinary divergence. By extrapolation, asymmetrical deviations from a general expectation of cross-species compatibility, such as in the case of Sir4, may signal situations of uncommon interest.

The studies presented here capitalized on the extraordinary genetic properties of interspecies hybrids to tease out important dimensions to the evolution and structure of silent chromatin in yeast. Although silencing behavior in these yeast hybrids was rather unusual, some type of defect might have been anticipated from recent studies of hybrid sterility or lethality genes in *Drosophila*, which have implicated rapidly evolving heterochromatin proteins as key factors contributing to interspecies genetic incompatibility [55],[56]. There is presently no reason to believe that *SIR1* or *SIR4* play roles in the post-zygotic genetic incompatibility between budding yeast species. It is notable, however, that in budding yeast multiple regulatory sites mediating silencing have rapidly evolved in a phylogenetically asymmetrical fashion along with a set of divergent silencing proteins, paralleling observations of rapid evolution in *Drosophila* heterochromatin [55],[57],[58]. It will be of great interest to determine whether the similar patterns of heterochromatin evolution in these distant taxa reflect similar underlying evolutionary processes.

### Implications for the Mechanism of Heterochromatin Formation

The unprecedented resolution of Sir4 distribution provided by ChIP-Seq methods calls into question earlier models for silenced chromatin assembly, and in particular the so-called mechanism of spreading (reviewed in [7],[59]). In the common view, Sir protein recruitment to the silencers or to telomeres allows the deacetylation of H4K16-Ac on adjacent histones, creating new binding sites for additional Sir protein complexes, with sequential cycles of deacetylation and binding leading to spreading of Sir-protein complexes across all nucleosomes in silenced chromatin. The strikingly uneven distribution of Sir4 at *HML* and *HMR* (Figure 4A, note *y*-axes), and at the telomeres (Figure 4B), as shown here and in *K. lactis* [60], is not entirely inconsistent with the common view of heterochromatin spreading, but is in no way anticipated by it. Clearly, high-resolution characterization of all Sir proteins by ChIP-Seq has the potential to force substantial revision or replacement of the current view.

## Materials and Methods

### Yeast Strain Construction and Genetic Manipulations

All *S. cerevisiae* strains were of the W303 background. Generation of marked *S. bayanus* strains from type strain CBS 7001 has been described [61]. All yeast strains were cultured at 25°C in standard yeast media. One-step gene replacement and C-terminal 13xMyc tag integration have been described previously [36],[62], and these genetic manipulations were performed identically for *S. bayanus*, *S. cerevisiae*, and *S. cerevisiae/S. bayanus* hybrids. The *HMR::URA3* reporter strains were constructed independently in *S. cerevisiae sir4Δ* and *S. bayanus sir4Δ* haploid strains, wherein the *HMR* **a**1 ORF was replaced with the *K. lactis URA3* ORF by PCR-based gene targeting, leaving the *HMR* **a**1 promoter intact. For most experiments, interspecies hybrids were made by crossing *S. bayanus MAT*α *HMR::URA3* strains (wild-type, *sir4Δ*, or *SIR4-13xMyc*) to *S. cerevisiae MAT* **a** strains (*wild-type*, *sir4Δ*, or *SIR4-13xMyc*). For *ORC1*, *RAP1*, and *ABF1* heterozygote analysis, gene targeting was performed directly in hybrid diploids or *S. bayanus* diploids. Three independent transformants were analyzed in all cases. *Sc-SIR4-13xMyc* and *Sb-SIR4-13xMyc* alleles were shown to be functional by two independent silencing assays in each case: by mating ability in *S. cerevisiae SIR4-13xMyc* and *S. bayanus SIR4-13xMyc* haploid strains and by FOA resistance in hybrid diploids bearing the appropriate *HMR::URA3* reporter (unpublished data).

The *Sc::(Sb-HMR::URA3)* replacement allele (Figure 6A) was generated in two steps. The *Sb-HMR::URA3* cassette plus 1 kb of leftward-flanking sequence was PCR-amplified out of the *S. bayanus* genome, and the PCR product was used to replace the syntenic portion of *Sc-HMR* (including the *E* silencer) in *S. cerevisiae sir4Δ* strains. A *HygMX* marker was then targeted into the *S. bayanus* genome 3 kb to the right of *Sb-HMR*. The entire rightward-flanking 3 kb region plus the *HygMX* marker was PCR-amplified out of the *S. bayanus* genome, and the PCR product was used to replace the syntenic portion of *Sc-HMR* in the *S. cerevisiae* genome (including the *I* silencer). The *Sc::(Sb-HMR::URA3)* replacement allele therefore included a total of 5.5 kb of *Sb-HMR* sequence, plus the 1.7 kb *HygMX* marker.

To construct the *SIR4* replacement alleles, the *Sc-SIR4* and *Sb-SIR4* genes were separately cloned into the yeast plasmid pRS315 [63] such that the *LEU2* marker was 5′ of, and in opposite orientation to, each *SIR4* gene. Each *SIR4* gene plus the *LEU2* marker was PCR-amplified from each plasmid. The *LEU2-Sc-SIR4* PCR product was used to replace the *URA3* marker at the *Sb-SIR4* locus in an *S. bayanus sir4Δ::URA3 leu2* strain; likewise, the *LEU2-Sb-SIR4* PCR product was targeted into the *Sc-SIR4* locus in an *S. cerevisiae sir4Δ::URA3 leu2* strain. The integrated *Sc-SIR4* gene was shown to silence *Sc-HMR::URA3* in hybrids (Figure 3A), and the integrated *Sb-SIR4* gene was shown to silence *Sb-HMR::URA3* in hybrids (Figure 6C) and *Sc-HML* and *Sc-HMR* in *S. cerevisiae* strains (Zill et al. in preparation). The expression level of each *SIR4* replacement allele was determined by quantitative RT-PCR (Figure S2A).

### Silencing Reporter Assays

Assays of yeast strain growth on FOA and CSM/-Ura media were performed using standard “frogging” techniques. Briefly, for each strain, a 10-fold dilution series of yeast cells at an approximate density of 4×10^7^/mL was spotted onto each plate. For Figures 2, 3A, 6, and 8A plates were photographed after 2 d for YPD, and after 3 d for FOA and CSM/-Ura. For Figure 8B, plates were photographed after 3 d for all media. For Figure 9B, plates were photographed after 3 d for FOA and YPD, and after 5 d for CSM/-Ura. We note that some changes in silencing could be seen only on FOA and not on CSM/-Ura. Incomplete silencing of the *HMR* **a**1 promoter likely led to heterogeneous expression states within the population of cells, with some remaining silent while others were expressed [64].

### RNA and Protein Analysis

RNA isolation was performed using the hot-phenol method [65]. Total RNA was digested with Amplification grade DNase I (Invitrogen) and purified using the RNeasy MinElute kit (Qiagen). cDNA was synthesized using the SuperScript III First-Strand Synthesis System for RT-PCR and oligo(dT) primer (Invitrogen). Quantitative PCR on cDNA was performed using an MX3000P machine (Stratagene) and the DyNAmo HS SYBR Green qPCR kit (NEB). Amplification values for all primer sets were normalized to actin (*ACT1*) or *SEN1* cDNA amplification values. Samples were analyzed in triplicate from three independent RNA preparations.

Yeast whole cell extracts were prepared using 20% TCA and solubilized in SDS loading buffer plus 100 mM Tris base. SDS-PAGE and immunoblotting were performed using standard procedures and the LiCOR imaging system. Anti-c-Myc antibody from rabbit (Sigma, Cat. No. C3956) was used to detect Myc-tagged Sir4 and Abf1 proteins. Mouse anti-Pgk1 antibody (Invitrogen, Cat. No. 459250) was used to verify equal loading. The *S. cerevisiae* Orc5-HA strain derivation has been described [66].

All chromatin immunoprecipitations (Sir4-Myc, Orc5-HA, Abf1-Myc) were performed as described [67], using formaldehyde cross-linking of log phase cultures for 1 h at room temperature. IPs were performed overnight at 4°C using Anti-c-Myc-Agarose (Sigma, Cat. No. A7470) and Anti-HA-Agarose (Sigma, Cat. No. A2095). Quantitative PCR was performed as described above.

### ChIP-Seq Analysis of Sir4 in *S. cerevisiae/S. bayanus* Hybrids

#### Library preparation and sequencing

ChIP-sequencing libraries were prepared from chromatin input and Sir4 precipitate fractions as per the Illumina paired-end and ChIP-Seq protocols, with modifications as per [68],[69]. Specifically, for both input and IP chromatin fractions, 1 µg of DNA was used for library construction; melting of gel slices for size selection was performed at room temperature to prevent loss of AT-rich sequences; and 18 cycles of PCR were performed to enrich adapter-ligated fragments. Library size and quality were verified by Bioanalyzer and quantitative PCR analysis. Insert size, excluding adapter sequences, averaged 320 bp for all libraries. Each input or IP library was loaded onto a single lane on a flow cell, and was sequenced by 36 bp paired-end reads on the Illumina Genome Analyzer II. All sequencing reads have been deposited in the Short Read Archive at NCBI under accession number SRP004163.

#### Data analysis

Paired-end reads were mapped to an *S. cerevisiae/S. bayanus* hybrid genome, made by concatenating the two species' genomes into a single FASTA file, using the MAQ software [70]. Every base of the hybrid genome was assigned the total number of sequence reads mapping to it, done separately for the input and IP reads. Median read counts were calculated for each set of reads in 100 bp windows, sliding along each chromosome or contig in 50 bp steps [71]. Median genome-wide coverage ranged 25–41× across all input samples and 8–13× across all IP samples. Correlation coefficients of median coverage values for each 100 bp window were determined for all pairs of datasets using R. Correlation coefficients were R = 0.96–0.98 for all possible pairs of input samples and R = 0.98–0.99 for all IP samples.

For select telomeric regions, the IP/input ratio of read counts was determined for each base and subsequently plotted versus chromosome position (Figures 4B and 5B). Binding of Sc-Sir4 and Sb-Sir4 to specific *S. cerevisiae* telomeres, and to putative *S. bayanus* subtelomeric regions, was confirmed using the peak-calling software MACS [72], which allowed determination of statistical confidence by modeling IP/Input background noise across the genome. For all other analyses, IP/Input ratios were calculated for all 100 bp sliding windows covering select 600 bp silenced regions: the *HMR-E* and *HML-E* silencers of both species, the *Sc-HMR* **a**1 and *Sb-HMR::URA3* ORFs, and control regions. The mean IP/Input ratio across these windows was determined for each 600 bp region (Table 1). For *Sc-HMR-E*, *Sb-HMR-E*, and *Sb-HMR::URA3*, the normalized (mean IP/Input, *query region*)∶(mean IP/Input, *control region*) ratio was plotted (Figure 4A).

### Evolutionary Analyses

Orthologous *S. cerevisiae* and *S. bayanus* genes were identified on the basis of sequence similarity and syntenic context (D. Scannell and M. B. Eisen, unpublished). Percent identities between 4,981 orthologous *S. cerevisiae* and *S. bayanus* proteins were then obtained by running BLASTP [73] with default parameters, imposing an E-value cutoff of 1×10^−5^, harvesting percent identities for each HSP and calculating a length-weighted average. This will necessarily lead to some underestimation of the true divergence between protein pairs, but it is unlikely that the rank order of divergences among pairs would be significantly affected.

For PAML and sliding window analyses, protein alignments were produced using FSA with default parameters [74], and DNA alignments were obtained by back translation with RevTrans [75]. All site and branch models were fit using codeml in the PAML package [76]. To test for positive selection we compared model M8 to M7 using a χ^2^ test with two degrees-of-freedom. Posterior probabilities of ω>1 for individual codons were obtained from the Bayes Empirical Bayes output of M8 only. For the sliding window (dN/dS, or ω) analyses, a window size of 102 bp and a step-size of 3 bp were used. Only alignment windows without gaps were analyzed. For each window we used codeml to estimate a single ω using model M0 implemented in codeml. All other parameters were estimated from the data.

We estimated the level of selective constraint operating on *SIR4* on each branch of the *Saccharomyces sensu stricto* phylogeny by computing branch-specific ratios of non-synonymous to synonymous substitutions (dN/dS, or ω). Briefly, we performed protein-space alignments of orthologous *SIR4* coding sequences with FSA [74] and then used codeml in the PAML package [76] to fit a “free-ratio” model (model = 1, NSSites = 0) to the alignment and obtain independent estimates of ω for each branch.

## Supporting Information

Figure S1 **Cross-species complementation analysis of** ***sir2Δ*** **and** ***sir3Δ*** **mutations in** ***S. cerevisiae*** **/** ***S. bayanus*** **interspecies hybrids.** Top panel: Ten-fold serial dilutions of hybrid strains bearing a *URA3* reporter gene at the *S. cerevisiae HMR* locus (*Sc-HMR::URA3*) were grown on medium counter-selective for *URA3* expression (FOA), selective for *URA3* expression (CSM/-Ura), or rich medium (YPD). Genotypes of both species' *SIR2* or *SIR3* genes are indicated at left. Bottom panel: Hybrid strains bearing a *URA3* reporter gene at the *S. bayanus HMR* locus (*Sb-HMR::URA3*), with genotypes of both species' *SIR2* or *SIR3* genes indicated at left.
(1.75 MB TIF)

Figure S2 ***SIR4*** **expression analysis in** ***S. cerevisiae*** **,** ***S. bayanus*** **, and** ***S. cerevisiae/S. bayanus*** **interspecies hybrids.** (A) *Sc-SIR4* and *Sb-SIR4 RNA* analysis by quantitative RT-PCR. Amplification values for *SIR4* cDNA were normalized to those of actin (*ACT1*), as indicated in Methods. Left to right: *Sc-SIR4* expression in *S. cerevisiae* haploid (JRY4012); *Sb-SIR4* expression in *S. bayanus* haploid (JRY8822); expression of *Sc-SIR4* replacement allele in *S. bayanus* haploid (JRY9049); expression of *Sb-SIR4* replacement allele in *S. cerevisiae* haploid (JRY9027); expression of either the *Sc-SIR4* or *Sb-SIR4* allele in a hybrid diploid (JRY9054). Note that because equivalent amounts of total cDNA were added to all qRT-PCR reactions, the apparent expression levels of *Sc-SIR4* and *Sb-SIR4* in this hybrid diploid were expected to be 50% of their levels in haploids. Error bars show standard deviations (*n* = 3). (B) Sc-Sir4 and Sb-Sir4 protein expression analysis by immunoblot. Left panel: A hybrid diploid with no Myc tag (lane 1), and Sc-Sir4-myc expression in *S. cerevisiae* diploids (lanes 2 and 3) and hybrid diploids (lanes 4 and 5). Right panel: Sb-Sir4-myc expression in *S. bayanus* diploids (lanes 6 and 7) and hybrid diploids (lanes 8 and 9). Phosphoglucokinase (Pgk1) expression is shown as a loading control.
(0.85 MB TIF)

Figure S3 **Genetic interaction analysis of ORC, Rap1, and Abf1 silencing functions at** ***Sc-HMR*** **in interspecies hybrids.** Silencing of the *Sc-HMR::URA3* reporter gene in *S. cerevisiae/S. bayanus* hybrids each lacking a single allele of the *RAP1*, *ORC1*, or *ABF1* genes (dilutions, plating, and photography performed as in Figure 9B).
(2.20 MB TIF)

Figure S4 ***SIR4*** **tree and branch-specific estimates of the ratio of nonsynonymous to synonymous divergence (ω).** Branch lengths represent the number of substitutions per codon.
(0.31 MB TIF)

Table S1 **Complete genotypes of strains used in this study.** Unless otherwise indicated, all strains originated from this study. For all genes in *S. cerevisiae/S. bayanus* hybrids, allele configurations are given as *S. cerevisiae/S. bayanus*.
(0.12 MB DOC)

## Abbreviations

ORC: Origin Recognition Complex
Sir: Silent Information Regulator
