# Supplementary figures and images for: Co-Evolution of Transcriptional Silencing Proteins and the DNA Elements Specifying Their Assembly
Source: PLoS Biol. 2010 Nov 30;8(11):e1000550. doi: 10.1371/journal.pbio.1000550 (PMC2994660; doi:10.1371/journal.pbio.1000550)

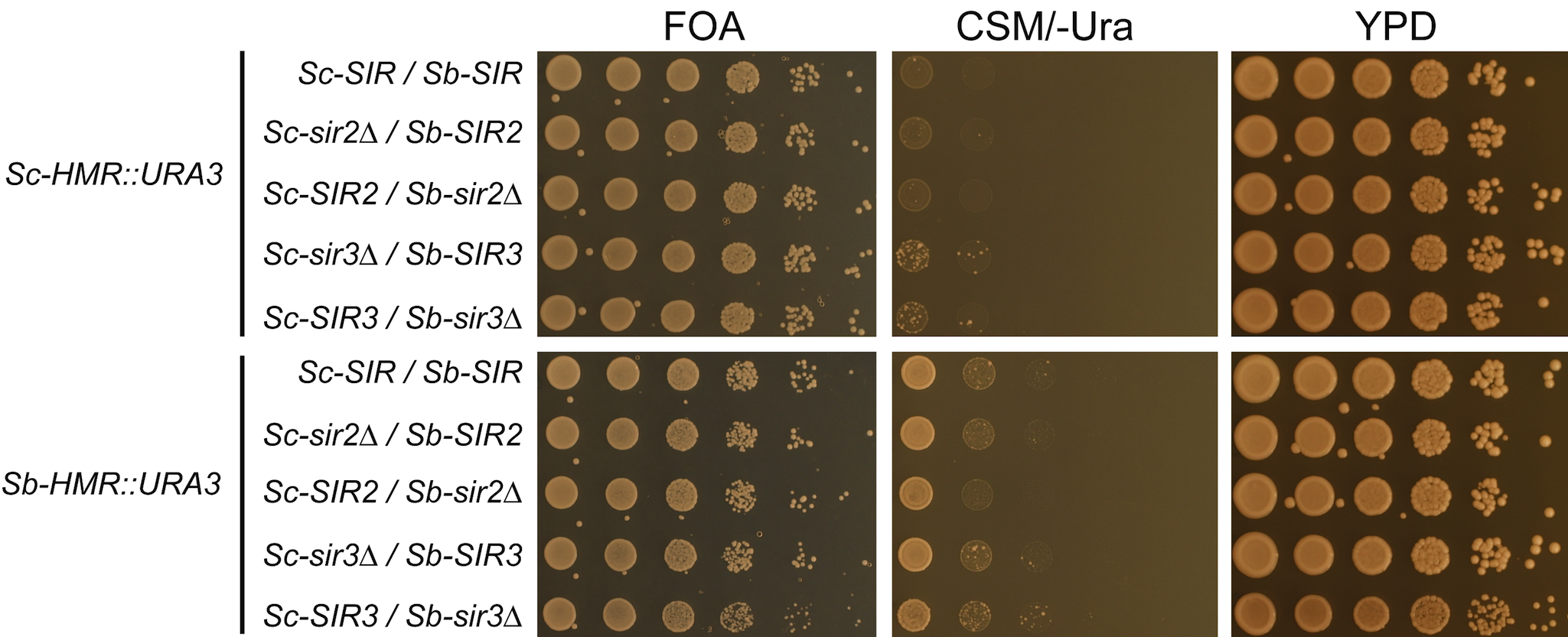

Supplement: Figure S1 — Cross-species complementation analysis of sir2Δ and sir3Δ mutations in S. cerevisiae / S. bayanus interspecies hybrids. Top panel: Ten-fold serial dilutions of hybrid strains bearing a URA3 reporter gene at the S. cerevisiae HMR locus (Sc-HMR::URA3) were grown on medium counter-selective for URA3 expression (FOA), selective for URA3 expression (CSM/-Ura), or rich medium (YPD). Genotypes of both species' SIR2 or SIR3 genes are indicated at left. Bottom panel: Hybrid strains bearing a URA3 reporter gene at the S. bayanus HMR locus (Sb-HMR::URA3), with genotypes of both species' SIR2 or SIR3 genes indicated at left. (1.75 MB TIF) [file pbio.1000550.s001.tif]

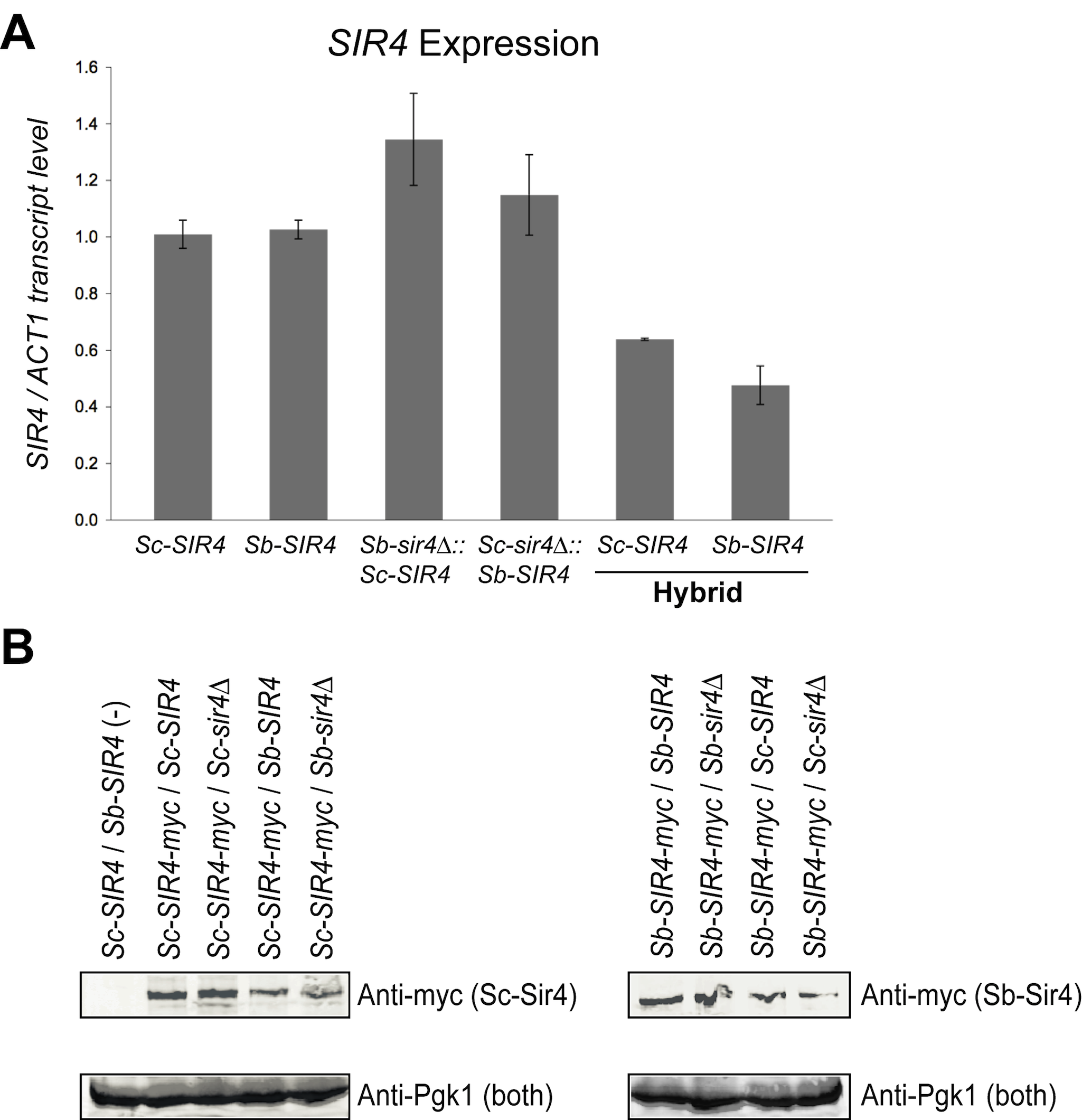

Supplement: Figure S2 — SIR4 expression analysis in S. cerevisiae , S. bayanus , and S. cerevisiae/S. bayanus interspecies hybrids. (A) Sc-SIR4 and Sb-SIR4 RNA analysis by quantitative RT-PCR. Amplification values for SIR4 cDNA were normalized to those of actin (ACT1), as indicated in Methods. Left to right: Sc-SIR4 expression in S. cerevisiae haploid (JRY4012); Sb-SIR4 expression in S. bayanus haploid (JRY8822); expression of Sc-SIR4 replacement allele in S. bayanus haploid (JRY9049); expression of Sb-SIR4 replacement allele in S. cerevisiae haploid (JRY9027); expression of either the Sc-SIR4 or Sb-SIR4 allele in a hybrid diploid (JRY9054). Note that because equivalent amounts of total cDNA were added to all qRT-PCR reactions, the apparent expression levels of Sc-SIR4 and Sb-SIR4 in this hybrid diploid were expected to be 50% of their levels in haploids. Error bars show standard deviations (n = 3). (B) Sc-Sir4 and Sb-Sir4 protein expression analysis by immunoblot. Left panel: A hybrid diploid with no Myc tag (lane 1), and Sc-Sir4-myc expression in S. cerevisiae diploids (lanes 2 and 3) and hybrid diploids (lanes 4 and 5). Right panel: Sb-Sir4-myc expression in S. bayanus diploids (lanes 6 and 7) and hybrid diploids (lanes 8 and 9). Phosphoglucokinase (Pgk1) expression is shown as a loading control. (0.85 MB TIF) [file pbio.1000550.s002.tif]

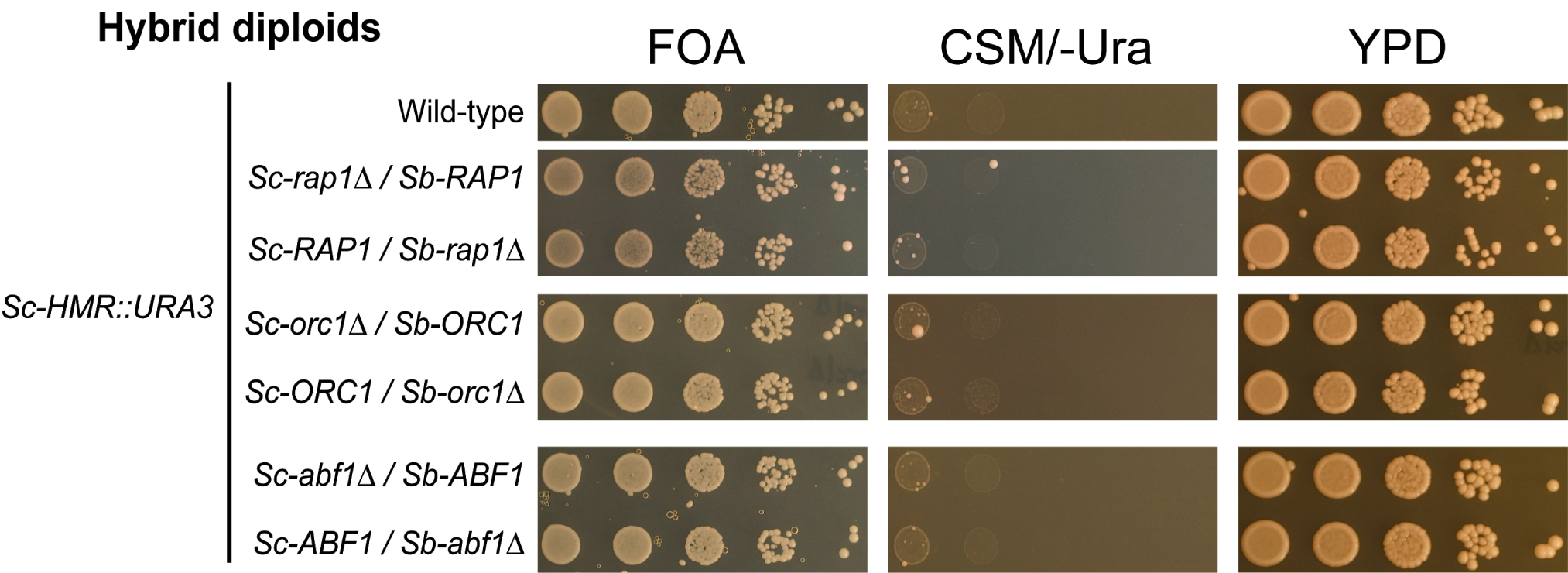

Supplement: Figure S3 — Genetic interaction analysis of ORC, Rap1, and Abf1 silencing functions at Sc-HMR in interspecies hybrids. Silencing of the Sc-HMR::URA3 reporter gene in S. cerevisiae/S. bayanus hybrids each lacking a single allele of the RAP1, ORC1, or ABF1 genes (dilutions, plating, and photography performed as in Figure 9B). (2.20 MB TIF) [file pbio.1000550.s003.tif]

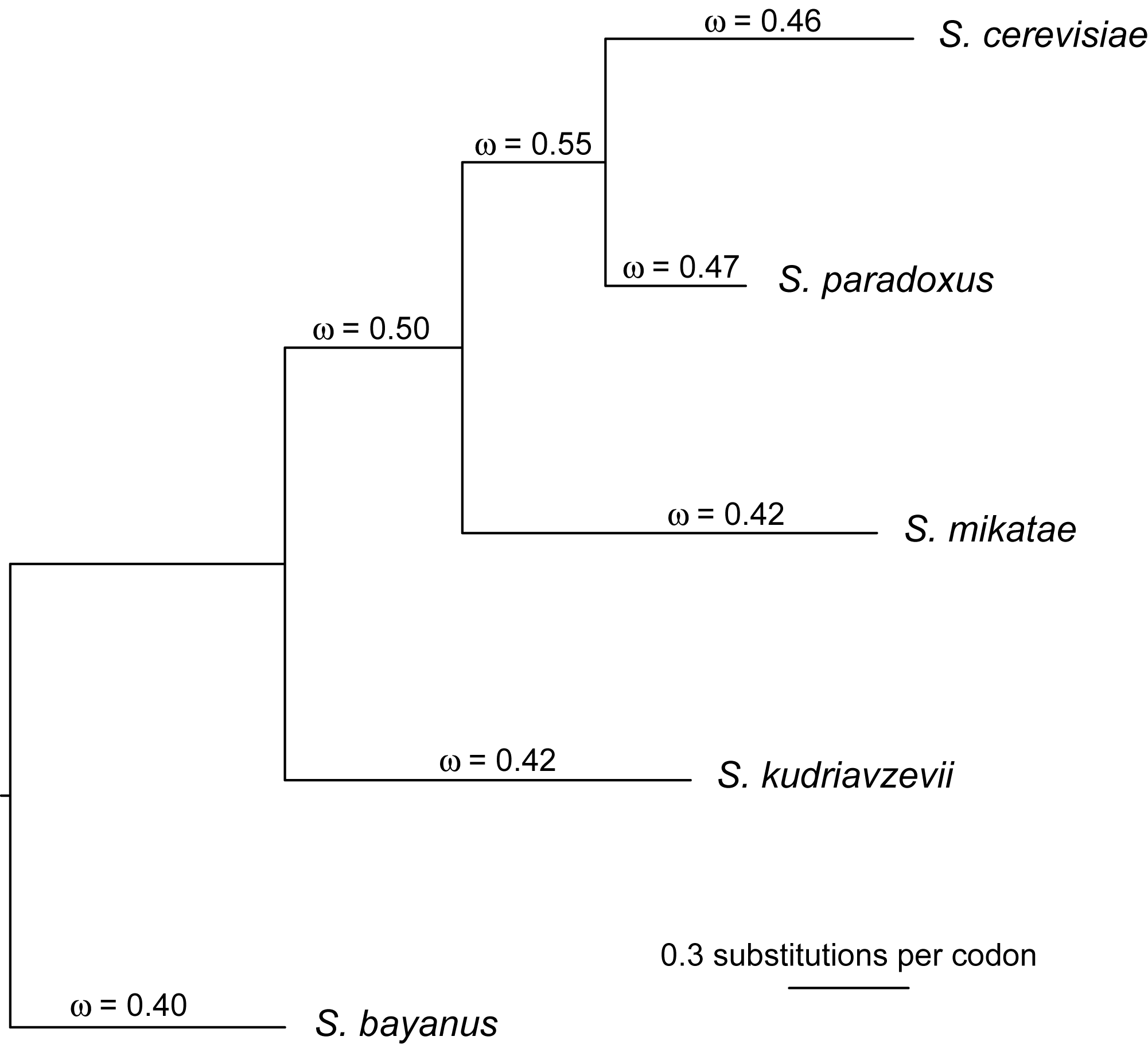

Supplement: Figure S4 — SIR4 tree and branch-specific estimates of the ratio of nonsynonymous to synonymous divergence (ω). Branch lengths represent the number of substitutions per codon. (0.31 MB TIF) [file pbio.1000550.s004.tif]
