# Supplementary material for: Co-Evolution of Transcriptional Silencing Proteins and the DNA Elements Specifying Their Assembly
Source: PLoS Biol. 2010 Nov 30;8(11):e1000550. doi: 10.1371/journal.pbio.1000550 (PMC2994660; doi:10.1371/journal.pbio.1000550)
Supplement: Table S1 — Complete genotypes of strains used in this study. Unless otherwise indicated, all strains originated from this study. For all genes in S. cerevisiae/S. bayanus hybrids, allele configurations are given as S. cerevisiae/S. bayanus. (0.12 MB DOC) [file pbio.1000550.s005.doc]

| **Strain** | **Species** | **Genotype** |
| --- | --- | --- |
| JRY4012 | *S. cerevisiae* (W303) | *MAT***a** *his3 leu2 lys2 trp1 ura3 can1* (Source: R. Rothstein) |
| JRY4013 | *S. cerevisiae* | *MAT*α *his3 leu2 lys2 trp1 ura3 can1* (Source: R. Rothstein) |
| JRY8821 | *S. cerevisiae* | *MAT*α *HMR::URA3 ade2* *his3 leu2 lys2 trp1 ura3* |
| JRY8676 | *S. cerevisiae* | *MAT*α *HMR::URA3 sir4Δ::HIS3* *ade2* *his3 leu2 trp1 ura3* |
| JRY9025 | *S. cerevisiae* | *MAT***a** *hmlΔ::KanMX sir4Δ::HIS3* *his3 leu2 lys2 trp1 ura3* |
| JRY9026 | *S. cerevisiae* | *MAT***a** *SIR4-13xMyc::KanMX* *his3 leu2 lys2 trp1 ura3* |
| JRY9027 | *S. cerevisiae* | *MAT***a** *sir4Δ::LEU2-Sb-SIR4 his3 leu2 lys2 trp1 ura3* |
| JRY9028 | *S. cerevisiae* | *MAT*α *sir4Δ::LEU2-Sb-SIR4 his3 leu2 lys2 trp1 ura3* |
| JRY9029 | *S. cerevisiae* | *MAT*α *Sc::(Sb-HMR::URA3) sir4Δ::KanMX* *ade2* *his3 leu2 trp1 ura3* |
| JRY9030 | *S. cerevisiae* | *MAT***a**/α *HMR/Sc::(Sb-HMR::URA3) SIR4/sir4Δ::KanMX* (JRY4012 x JRY9029) |
| JRY9031 | *S. cerevisiae* | *MAT*α *Sc::(Sb-HMR::URA3) Sc-SIR4 his3 leu2 trp1 ura3* |
| JRY9032 | *S. cerevisiae* | *MAT*α *Sc::(Sb-HMR::URA3) Sc-sir4Δ::LEU2-Sb-SIR4 his3 leu2 lys2 trp1 ura3* |
| JRY9033 | *S. cerevisiae* | *MAT*α *Sc::(Sb-HMR::URA3) Sc-SIR4 sir1Δ::TRP1 his3 leu2 lys2 trp1 ura3* |
| JRY9034 | *S. cerevisiae* | *MAT*α *Sc::(Sb-HMR::URA3) Sc-sir4Δ::LEU2-Sb-SIR4 sir1Δ::TRP1 his3 leu2 lys2 trp1 ura3* |
| JRY9035 | *S. cerevisiae* | *MAT*α *ORC5-HA::KanMX his3 leu2 trp1 ura3* |
| JRY9036 | *S. cerevisiae* | *MAT*α *Sc-sir4Δ::LEU2-SbSIR4 ORC5-HA::KanMX his3 leu2 lys2 trp1 ura3* |
| JRY9037 | *S. cerevisiae* | *MAT*α *Sc::(Sb-HMR::URA3) ORC5-HA::KanMX his3 leu2 trp1 ura3* |
| JRY9038 | *S. cerevisiae* | *MAT*α *Sc::(Sb-HMR::URA3) Sc-sir4Δ::LEU2-SbSIR4 ORC5-HA::KanMX his3 leu2 trp1 ura3* |
| JRY9039 | *S. cerevisiae* | *MAT*α *ABF1-13xMyc::KanMX* (JRY4013) |
| JRY9040 | *S. cerevisiae* | *MAT*α *sir4Δ::LEU2-SbSIR4 ABF1-13xMyc::KanMX* (JRY9029) |
| JRY9041 | *S. cerevisiae* | *MAT*α *Sc::(Sb-HMR::URA3) ABF1-13xMyc::KanMX* (JRY9031) |
| JRY9042 | *S. cerevisiae* | *MAT*α *Sc::(Sb-HMR::URA3) sir4Δ::LEU2-SbSIR4 ABF1-13xMyc::KanMX* (JRY9032) |
| JRY8822 | *S. bayanus* (CBS 7001) | *MAT***a** *hoΔ::NatMX* *lys2 ura3* |
| JRY8819 | *S. bayanus* | *MAT*α *HMR::URA3 ade2* *his3 lys2 ura3* |
| JRY9043 | *S. bayanus* | *MAT*α *HMR::URA3 sir4Δ::KanMX ade2* *ura3* |
| JRY8820 | *S. bayanus* | *MAT***a** *hmlΔ::S.p.his5 sir4Δ::KanMX his3 lys2 ura3* |
| JRY9044 | *S. bayanus* | *MAT***a** *SIR4-13xMyc::KanMX* *lys2 ura3* |
| JRY9045 | *S. bayanus* | *MAT*α *HMR::URA3* *SIR4-13xMyc::KanMX* *ade2 his3* *lys2 ura3* |
| JRY9046 | *S. bayanus* | *MAT***a**/α *HMR/HMR::URA3 SIR4/SIR4* (JRY8822 x JRY8819) |
| JRY9047 | *S. bayanus* | *MAT***a**/α *HMR/HMR::URA3 SIR4/sir4Δ::KanMX* (JRY8822 x JRY9043) |
| JRY9048 | *S. bayanus* | *MAT***a**/α *hmlΔ::S.p.his5/HML* *HMR/HMR::URA3 sir4Δ::KanMX/sir4Δ::KanMX*  (JRY8820 x JRY9043) |
| JRY9049 | *S. bayanus* | *MAT***a** *sir4Δ::LEU2-Sc-SIR4 hoΔ::KanMX leu2 lys2 trp1 ura3* |
| JRY9050 | *S. bayanus* | *MAT*α *HMR::URA3* *sir4Δ::LEU2-Sc-SIR4 leu2 lys2 trp1 ura3* |
| JRY9051 | *S. bayanus* | *MAT*α *HMR::URA3* *sir4Δ::LEU2-Sc-SIR4 ade2 his3 leu2 lys2 ura3* |
| JRY9052 | *S. bayanus* | *MAT***a***/*α *HMR/HMR::URA3* *RAP1/rap1Δ::HygMX* (JRY9046) |
| JRY9053 | *S. bayanus* | *MAT***a***/*α *HMR/HMR::URA3* *ORC1/orc1Δ::HygMX* |
| JRY9054 | *S. cerevisiae/S. bayanus* | *MAT***a**/α *Sc-HMR/Sb-HMR::URA3 Sc-SIR4/Sb-SIR4* (JRY4012 x JRY8819) |
| JRY9055 | *S. cerevisiae/S. bayanus* | *MAT***a**/α *Sc-HMR/Sb-HMR::URA3 Sc-SIR4/Sb-sir4Δ::KanMX* (JRY4012 x JRY9043) |
| JRY9056 | *S. cerevisiae/S. bayanus* | *MAT***a**/α *Sc-hmlΔ::KanMX/Sb-HML Sc-HMR/Sb-HMR::URA3 Sc-sir4Δ::HIS3/Sb-SIR4*  (JRY9025 x JRY8819) |
| JRY9057 | *S. cerevisiae/S. bayanus* | *MAT***a**/α *Sc-hmlΔ::KanMX/Sb-HML Sc-HMR/Sb-HMR::URA3 Sc-sir4Δ::HIS3/Sb-sir4Δ::KanMX*  (JRY9025 x JRY9043) |
| JRY9058 | *S. cerevisiae/S. bayanus* | *MAT*α*/***a** *Sc-HMR::URA3/Sb-HMR Sc-SIR4/Sb-SIR4* (JRY8821 x JRY8822) |
| JRY9059 | *S. cerevisiae/S. bayanus* | *MAT*α*/***a** *Sc-HML/Sb-hmlΔ::S.p.his5* *Sc-HMR::URA3/Sb-HMR Sc-SIR4/Sb-sir4Δ::KanMX*  (JRY8821 x JRY8820) |
| JRY9060 | *S. cerevisiae/S. bayanus* | *MAT*α*/***a** *Sc-HMR::URA3/Sb-HMR Sc-sir4Δ::HIS3/Sb-SIR4* (JRY8676 x JRY8822) |
| JRY9061 | *S. cerevisiae/S. bayanus* | *MAT*α*/***a** *Sc-HML/Sb-hmlΔ::S.p.his5* *Sc-HMR::URA3/Sb-HMR Sc-sir4Δ::HIS3/Sb-sir4Δ::KanMX* (JRY8676 x JRY8820) |
| JRY9062 | *S. cerevisiae/S. bayanus* | *MAT***a**/α *Sc-HMR/Sb-HMR::URA3 Sc-SIR4-13xMyc::KanMX/Sb-sir4Δ::LEU2-Sc-SIR4* (JRY9026 x JRY9051) |
| JRY9063 | *S. cerevisiae/S. bayanus* | *MAT***a**/α *Sc-HMR/Sb-HMR::URA3 Sc-sir4Δ::LEU2-Sb-SIR4/Sb-SIR4-13xMyc::KanMX* (JRY9027 x JRY9045) |
| JRY9064 | *S. cerevisiae/S. bayanus* | *MAT***a**/α *Sc-HMR/Sb-HMR::URA3 Sc-SIR4-13xMyc::KanMX/Sb-SIR4* (JRY9026 x JRY8819) |
| JRY9065 | *S. cerevisiae/S. bayanus* | *MAT***a**/α *Sc-HMR/Sb-HMR::URA3 Sc-SIR4/Sb-SIR4-13xMyc::KanMX* (JRY4012 x JRY9045) |
| JRY9066 | *S. cerevisiae/S. bayanus* | *MAT*α/**a** *Sc-HMR::URA3/Sb-HMR Sc-sir4Δ::HIS3/sir4Δ::LEU2-Sc-SIR4*  (JRY8676 x JRY9049) |
| JRY9067 | *S. cerevisiae/S. bayanus* | *MAT*α*/***a** *Sc::(Sb-HMR::URA3)/Sb-HMR Sc-sir4Δ::KanMX/Sb-SIR4* (JRY9029 x JRY8822) |
| JRY9068 | *S. cerevisiae/S. bayanus* | *MAT***a**/α *Sc-HMR/Sb-HMR::URA3 Sc-sir4Δ::LEU2-Sb-SIR4/Sb-sir4Δ::KanMX*  (JRY9027 x JRY9043) |
| JRY9069 | *S. cerevisiae/S. bayanus* | *MAT***a***/*α *Sc-HMR/Sb-HMR::URA3 Sc-rap1Δ::HygMX/Sb-RAP1* (JRY9054) |
| JRY9070 | *S. cerevisiae/S. bayanus* | *MAT***a***/*α *Sc-HMR/Sb-HMR::URA3 Sc-RAP1/Sb-rap1Δ::HygMX* |
| JRY9071 | *S. cerevisiae/S. bayanus* | *MAT***a***/*α *Sc-HMR/Sb-HMR::URA3* *Sc-orc1Δ::HygMX/Sb-ORC1* |
| JRY9072 | *S. cerevisiae/S. bayanus* | *MAT***a***/*α *Sc-HMR/Sb-HMR::URA3* *Sc-ORC1/Sb-orc1Δ::HygMX* |
| JRY9073 | *S. cerevisiae/S. bayanus* | *MAT***a***/*α *Sc-HMR/Sb-HMR::URA3* *Sc-abf1Δ::HygMX/Sb-ABF1* |
| JRY9074 | *S. cerevisiae/S. bayanus* | *MAT***a***/*α *Sc-HMR/Sb-HMR::URA3* *Sc-ABF1/Sb-abf1Δ::HygMX* |
| JRY9165 | *S. cerevisiae/S. bayanus* | *MAT***a***/*α *Sc-HMR::URA3/Sb-HMR Sc-rap1Δ::HygMX/Sb-RAP1* (JRY9058) |
| JRY9166 | *S. cerevisiae/S. bayanus* | *MAT***a***/*α *Sc-HMR::URA3/Sb-HMR Sc-RAP1/Sb-rap1Δ::HygMX* |
| JRY9167 | *S. cerevisiae/S. bayanus* | *MAT***a***/*α *Sc-HMR::URA3/Sb-HMR* *Sc-orc1Δ::HygMX/Sb-ORC1* |
| JRY9168 | *S. cerevisiae/S. bayanus* | *MAT***a***/*α *Sc-HMR::URA3/Sb-HMR* *Sc-ORC1/Sb-orc1Δ::HygMX* |
| JRY9169 | *S. cerevisiae/S. bayanus* | *MAT***a***/*α *Sc-HMR::URA3/Sb-HMR* *Sc-abf1Δ::HygMX/Sb-ABF1* |
| JRY9170 | *S. cerevisiae/S. bayanus* | *MAT***a***/*α *Sc-HMR::URA3/Sb-HMR* *Sc-ABF1/Sb-abf1Δ::HygMX* |
| JRY9164 | *S. cerevisiae/S. bayanus* | *MAT***a**/α *Sc::(Sb-HMR::URA3)/Sb-HMR Sc-sir4Δ::LEU2-Sb-SIR4/Sb-SIR4* |
| JRY9146 | *S. cerevisiae/S. bayanus* | *MAT***a**/α *Sc-HMR/Sb-HMR::URA3 Sc-sir2Δ::HygMX/Sb-SIR2* (JRY9054) |
| JRY9147 | *S. cerevisiae/S. bayanus* | *MAT***a**/α *Sc-HMR/Sb-HMR::URA3 Sc-SIR2/Sb-sir2Δ::HygMX* (JRY9054) |
| JRY9148 | *S. cerevisiae/S. bayanus* | *MAT***a**/α *Sc-HMR/Sb-HMR::URA3 Sc-sir3Δ::HygMX/Sb-SIR3* (JRY9054) |
| JRY9149 | *S. cerevisiae/S. bayanus* | *MAT***a**/α *Sc-HMR/Sb-HMR::URA3 Sc-SIR3/Sb-sir3Δ::HygMX* (JRY9054) |
| JRY9150 | *S. cerevisiae/S. bayanus* | *MAT*α*/***a** *Sc-HMR::URA3/Sb-HMR Sc-sir2Δ::HygMX/Sb-SIR2* (JRY9058) |
| JRY9151 | *S. cerevisiae/S. bayanus* | *MAT*α*/***a** *Sc-HMR::URA3/Sb-HMR Sc-SIR2/Sb-sir2Δ::HygMX* (JRY9058) |
| JRY9152 | *S. cerevisiae/S. bayanus* | *MAT*α*/***a** *Sc-HMR::URA3/Sb-HMR Sc-sir3Δ::HygMX/Sb-SIR3* (JRY9058) |
| JRY9153 | *S. cerevisiae/S. bayanus* | *MAT*α*/***a** *Sc-HMR::URA3/Sb-HMR Sc-SIR3/Sb-sir3Δ::HygMX* (JRY9058) |
